# Conformational dynamics of complement protease C1r inhibitor proteins from Lyme disease– and relapsing fever–causing spirochetes

**DOI:** 10.1016/j.jbc.2023.104972

**Published:** 2023-06-27

**Authors:** Sourav Roy, Charles E. Booth, Alexandra D. Powell-Pierce, Anna M. Schulz, Jon T. Skare, Brandon L. Garcia

**Affiliations:** 1 Department of Microbiology and Immunology, Brody School of Medicine, East Carolina University, Greenville, North Carolina, USA; 2 Department of Microbial Pathogenesis and Immunology, School of Medicine, Texas A&M University, Bryan, Texas, USA

**Keywords:** molecular dynamics simulations, complement inhibitors, Lyme disease, relapsing fever, complement C1r, BBK32

## Abstract

Borrelial pathogens are vector-borne etiological agents known to cause Lyme disease, relapsing fever, and *Borrelia miyamotoi* disease. These spirochetes each encode several surface-localized lipoproteins that bind components of the human complement system to evade host immunity. One borrelial lipoprotein, BBK32, protects the Lyme disease spirochete from complement-mediated attack *via* an alpha helical C-terminal domain that interacts directly with the initiating protease of the classical complement pathway, C1r. In addition, the *B. miyamotoi* BBK32 orthologs FbpA and FbpB also inhibit C1r, albeit *via* distinct recognition mechanisms. The C1r-inhibitory activities of a third ortholog termed FbpC, which is found exclusively in relapsing fever–causing spirochetes, remains unknown. Here, we report the crystal structure of the C-terminal domain of *Borrelia hermsii* FbpC to a limiting resolution of 1.5 Å. We used surface plasmon resonance and assays of complement function to demonstrate that FbpC retains potent BBK32-like anticomplement activities. Based on the structure of FbpC, we hypothesized that conformational dynamics of the complement inhibitory domains of borrelial C1r inhibitors may differ. To test this, we utilized the crystal structures of the C-terminal domains of BBK32, FbpA, FbpB, and FbpC to carry out molecular dynamics simulations, which revealed borrelial C1r inhibitors adopt energetically favored open and closed states defined by two functionally critical regions. Taken together, these results advance our understanding of how protein dynamics contribute to the function of bacterial immune evasion proteins and reveal a surprising plasticity in the structures of borrelial C1r inhibitors.

Pathogenic bacteria must evade the immune system of their hosts in order to establish infection. To this end, many bacterial pathogens have evolved mechanisms to disrupt the powerful bacteriolytic activity and immune surveillance capacity of the complement system (1, 2, 3, 4, 5, 6, 7, 8). For example, the etiologic agents of borreliosis are master manipulators of the complement cascade due to the production of numerous anticomplement proteins on the bacterial surface (3, 9, 10). As such, the causative agent of Lyme disease, *Borreliella (Borrelia) burgdorferi*, has become a powerful model system for understanding mechanisms of microbial complement evasion (3, 9, 11, 12). Although less studied, several complement-targeting lipoproteins have also been described for other agents of borreliosis including tick-borne relapsing fever spirochetes (*i.e.*, *Borrelia hermsii*, *Borrelia turicatae*, etc.) (13, 14, 15, 16, 17), louse-borne relapsing fever spirochetes (*i.e.*, *Borrelia recurrentis*) (18, 19), and *Borrelia miyamotoi* spirochetes (20, 21, 22, 23).

An example of a borrelial surface-exposed lipoprotein that mediates complement inhibition is BBK32 (24, 25, 26, 27). BBK32 is produced *in vivo* and is important for optimal infection in a murine model of Lyme borreliosis (28, 29). BBK32 is a multifunctional protein that interacts with host glycosaminoglycans and fibronectin using separate intrinsically disordered N-terminal–binding sites (24, 26, 28, 29). BBK32 also blocks the classical pathway (CP) of the complement system by specifically binding and inhibiting the initiating protease, C1r, *via* its carboxy terminal alpha helical domain (*i.e.*, BBK32-C) (3, 30, 31, 32). BBK32-C forms a high-affinity interaction with the serine protease domain of human C1r and binds both zymogen and activated forms of C1r (30, 31). Small angle X-ray scattering and X-ray crystallography structures of the complex between fragments of human C1r and BBK32-C, coupled with extensive site-directed mutagenesis studies, further defined the protein–protein interface formed between BBK32-C and C1r (32). These studies revealed that BBK32 occludes the S1 subsite of the C1r active site with a cluster of residues located near a small loop that connects BBK32-C alpha helix 1 and alpha helix 2 (32). At this BBK32/C1r-binding site—which we will refer to as the “K1 site” here forward—a key arginine residue of BBK32 (*i.e.*, R248) was shown to be critical to both C1r-binding affinity and complement inhibitory activity (32).

A second BBK32/C1r-binding surface, which we term here the “K2 site”, involves BBK32 residues on alpha helix 5, typified by lysine 327 of BBK32 (K327). The precise inhibitory functions of the K2 site are less clear. However, the K2 site, which is ∼25 Å away from the K1 site, interfaces with nonactive site residues on a surface loop of C1r known as the B-loop. The importance of the K2 site is accentuated by the BBK32-K327A mutant, which exhibits an ∼150-fold decrease in C1r-binding affinity and significantly reduced complement inhibitory activity (32). Furthermore, natural mutations for another residue of the K2 site (*i.e.*, BBK32-E324) were identified in a homolog from avian-associated *Borrelia garinii* (*i.e.*, BGD19) that demonstrated significantly reduced activity against human complement (31). Nonetheless, in BBK32, only simultaneous mutation of both R248 and K327 within the K1 and K2 site, respectively, (*i.e.*, BBK32-R248A-K327A) resulted in a protein that lacked measurable C1r binding and caused a complete loss of serum resistance in a serum-sensitive *Borrelia burgdorferi* background (32). Thus, both the K1 and K2 sites each contribute significantly to the activity of BBK32 against human complement.

Relapsing fever spirochetes encode BBK32-like proteins that organize phylogenetically into three families known as FbpA, FbpB, and FbpC (20, 33). Whereas Lyme disease spirochetes encode a single BBK32 protein, relapsing fever and *B. miyamotoi* spirochetes encode either two or, in some cases, all three Fbp proteins (20, 33). The C1r-binding properties and inhibitory mechanisms of Fbp proteins have been characterized for FbpA and FbpB from *B. miyamotoi* (20). FbpA interacts with C1r and inhibits the CP in a similar manner to BBK32, including retaining the ability to bind both zymogen and active forms of C1r (20). Mutations to the homologous K1 and K2 site residues in FbpA (*i.e.*, FbpA-R264A-K343A) resulted in a protein that lost all C1r binding and complement inhibitory activity, which provided independent validation of the importance of these conserved sites. Interestingly, FbpB bound only to the active form of C1r, and the crystal structure of the C-terminal region of FbpB revealed significant deviations in the secondary structure of the FbpB K2 site relative to both BBK32 and FbpA (20). FbpB also lacks the conserved Lys residue in the K2 site that is shared between BBK32 and FbpA (*i.e.*, BBK32-K327/FbpA-K343) and is instead replaced with a small insertion loop that interrupts the fifth alpha helix. Furthermore, several residues on this K2 site loop in FbpB are apparently flexible, as evidenced by a lack of interpretable electron density for these residues in crystallographic experiments of FbpB (20).

In contrast to BBK32, FbpA, and FbpB, little is known about the C1r-inhibitory properties of FbpC proteins. However, FbpC proteins from *B. recurrentis* and *Borrelia duttonii* have been reported to be involved in complement evasion by specific binding of the endogenous complement regulators C1 esterase inhibitor (C1-INH) and C4b-binding protein (C4BP) (18). This protein was designated CihC, and a putative binding site for C1-INH and C4BP was mapped to residues 145 to 185 (18). In a follow up study, CihC/FbpC orthologs were studied in *B. hermsii*, *Borrelia parkeri*, and *B. turicatae* (34). While none of these proteins retained the C1-INH or C4BP-binding properties of *B. recurrentis* CihC/FbpC, four out of the five proteins bound specifically to fibronectin (34). Finally, *B. hermsii* FbpC from strain HS1 is known to be expressed during a murine model of infection (33). This protein, initially referred to as BHA007, was shown to bind to fibronectin and C4BP, and the authors proposed the BBK32/Fbp nomenclature which we have retained here (33). Collectively, these reports show that a subset of FbpC proteins use N-terminal–binding sites to recruit host complement regulators and serve as a fibronectin adhesin (18, 33, 34). However, none of these prior studies addressed the function of the C-terminal domain of FbpC.

In this study, we report the structural and functional characterization of the C-terminal region of a representative FbpC family member from *B. hermsii* strain HS1, termed FbpC-C hereafter. Our biochemical, microbiological, and biophysical studies demonstrate that *B. hermsii* FbpC is a potent inhibitor of the CP of human complement. Surprisingly, however, the high-resolution crystal structure of FbpC-C exhibited a striking difference in the conformation of the K1 site compared to BBK32, FbpA, and FbpB. Furthermore, a disordered stretch of eight residues in the FbpC-C K1 site suggested that this site may be dynamic. Based on these observations, we tested the hypothesis that protein dynamics play a previously unrecognized role in the structure and function of borrelial C1r inhibitors by carrying out detailed analysis of 1 μs molecular dynamics (MD) simulations in tandem with biochemical and biophysical assays utilizing site-directed mutants of FbpC-C.

## Results

### The crystal structure of the C-terminal domain of *B. hermsii* FbpC

**Figure 1:**
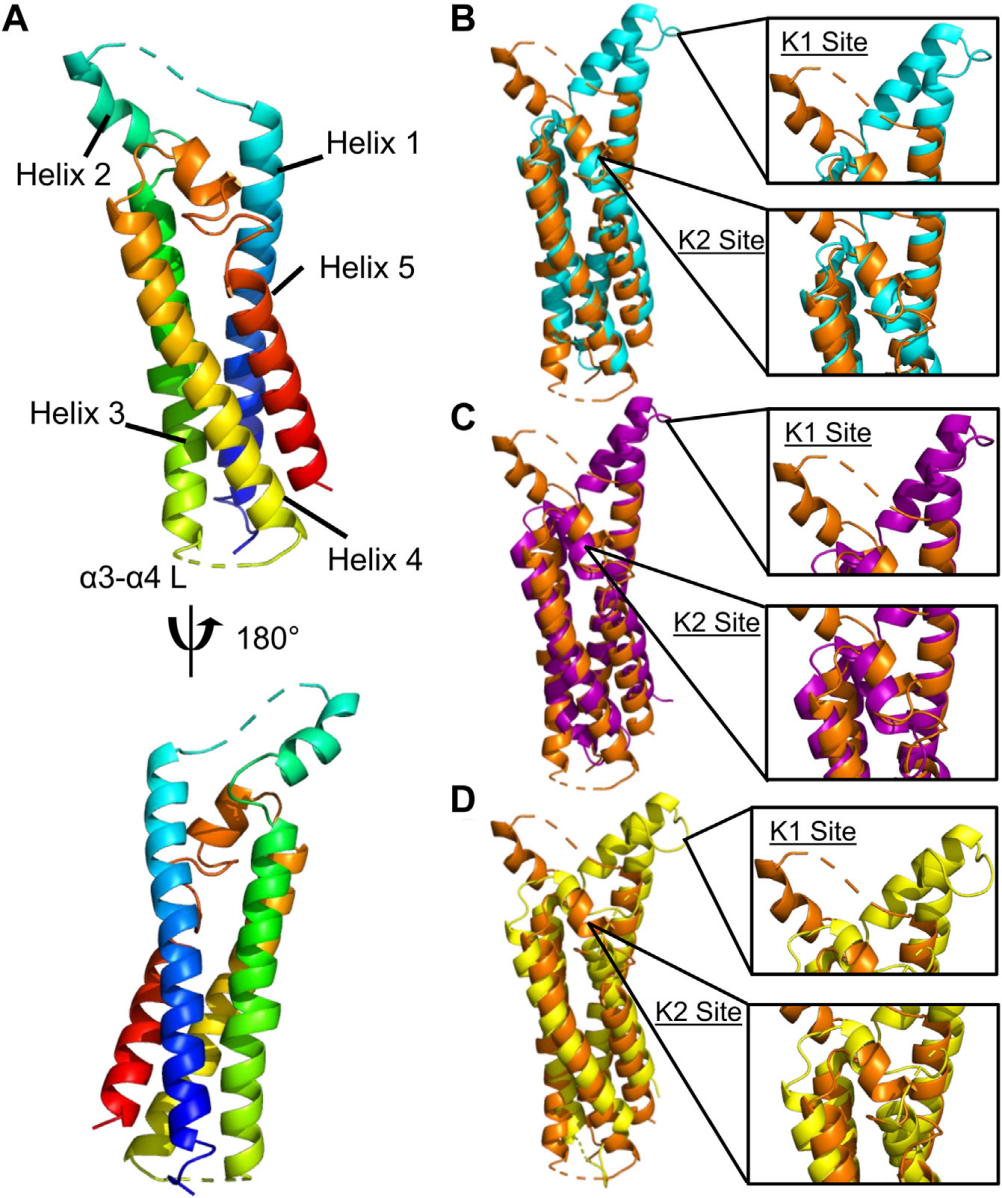
***Borrelia hermsii* FbpC-C crystal structure.***A*, the crystal structure of *B. hermsii* FbpC-C_212–374_ (from strain HS1) at a limiting resolution of 1.50 Å (PDB ID: 8EC3). The model is colored as a spectrum with the N-terminus in *blue* and C-terminus in *red*. The small loop connecting alpha helix 3 and alpha helix 4 is denoted “α3-α4 L”. Structural overlays of FbpC-C (*orange*) with (*B*) BBK32-C (*cyan*, PDB ID: 61NL), (*C*) FbpA-C (*purple*, PDB ID: 7RPR), and (*D*) FbpB-C (*yellow*, PDB ID: 7RPS). The K1 and K2 sites are defined as the areas of BBK32-C that interact with the S1 pocket and B-loop of C1r, respectively (32).

The crystal structures of the C-terminal complement inhibitory domains of *B. burgdorferi* BBK32-C (PDB ID: 6N1L), *B. miyamotoi* FbpA-C (PDB ID: 7RPR), and *B. miyamotoi* FbpB-C (PDB ID: 7RPS) have all been previously reported (20, 31). However, a representative structure of the C-terminal region of an FbpC family member remained elusive. We overcame this by identifying a crystallizable construct of *B. hermsii* strain HS1 FbpC corresponding to residues 212 to 374 (*i.e.*, FbpC-C) that diffracted to 1.50 Å limiting resolution (Figs. 1*A* and S1*A*, Table 1). Protein crystals of FbpC-C grew in space group P 2_1_ with a single molecule in the asymmetric unit. Initial phases were obtained by molecular replacement using a model derived from AlphaFold2 (35). Iterative rounds of refinement resulted in a final model (*R*_*work*_ = 17.7% and *R*_*free*_ = 19.8%, Table 1) that was deposited into the Protein Data Bank (PDB ID: 8EC3).

**Table 1:** Data collection and refinement statistics

Proteins	FbpC _(212–374)_
Data collection	
Space group	P 1 21 1
Cell dimensions	
a, b, c, Å	38.24, 46.56, 45.19
α, β, γ, ˚	90.00, 110.80, 90.00
Resolution, Å	31.28–1.50 (1.55–1.50)
*R*_pim_	0.032 (0.218)
*R*_meas_	0.087 (0.509)
*CC*_1/2_	0.989 (0.870)
I/*σ*I	19.4 (2.0)
Completeness, %	98.7 (89.2)
Redundancy	7.0 (4.2)
Refinement	
Resolution, Å	28.35-1.50 (1.55-1.50)
No. of reflections	22,687 (1796)
*R*_*work*_*/R*_*free*_	0.1770/0.1975
No. of nonhydrogen atoms	1393
Protein	1235
Water	157
B-factors	
Protein	30.38
Water	39.40
Rmsd	
Bond lengths, Å	0.010
Bond angles, °	1.05

Values in parentheses refer to the highest-resolution shell.

### Structural similarity between FbpC-C and BBK32-C/FbpA-C/FbpB-C

Analysis of the FbpC-C structure revealed that it shares a common four-helix bundle fold with BBK32-C, FbpA-C, and FbpB-C (Fig. 1) (20, 31). Our previous structural and biochemical studies identified two regions of BBK32-C that are critical for interaction with human C1r (31, 32). The first, denoted here as the “K1 site”, is found on a small loop that connects alpha helix 1 and alpha helix 2 and in BBK32, harbors a key arginine residue (*i.e.*, R248). A homologous arginine residue (*i.e.*, R264) was also shown to be important for C1r binding by FbpA-C, and FbpB-C presents R309 in a structurally similar position to both BBK32-C and FbpA-C (20). Intriguingly, a sequence alignment to BBK32-C, FbpA-C, and FbpB-C predict that a histidine residue in FbpC-C (*i.e.*, H252) takes the place of the key K1 site arginine residues noted above for BBK32-C/FbpA-C/FbpB-C (Fig. S2).

Surprisingly, the crystal structure of FbpC-C deviated from BBK32-C, FbpA-C, and FbpB-C in the conformation of K1 site in two major ways (Fig. 1). First, the relative angle of alpha helices 1-2-3 is ∼75° in FbpC-C, whereas this angle is ∼20 to 25° in BBK32-C, FbpA-C, and FbpB-C. The open conformation of the FbpC-C helix was not predicted in the AlphaFold2 model, where it instead adopts a closed conformation (∼23°) (Fig. S1*B*). While the AlphaFold2 model was characterized by overall very high confidence, as judged by predicted local distance difference test (pLDDT) ≥90 (35), we noted that both the K1 and K2 sites showed reduced pLDDT values, dipping near or below the low confidence threshold (*i.e.*, pLDDT ≤ 70) (Fig. S1*C*). The second notable feature of this region of the FbpC-C crystal structure was the lack of electron density corresponding to the residues that connect the first and second alpha helix (residues M_247_-RMSGHST_254_) (Figs. 1*A* and S1*A*). Flexible regions of proteins are often not visualized in crystal structures (36), and thus, this result suggests that FbpC-C harbors a longer flexible loop connecting the first and second alpha helix that was not found in the crystal structures of other BBK32/Fbp family members (20, 31).

A second site was shown to be critical for BBK32-C’s interaction with C1r and inhibition of complement, termed the “K2 site”, which involves surface residues presented near a kink in the C-terminal fifth alpha helix (32). In BBK32, the K2 site includes a key lysine residue that interacts with the B-loop of C1r (*i.e.*, K327) (32). A homologous lysine residue in FbpA-C (*i.e.*, K343) is also important for C1r binding and inhibition (20). In contrast, a homologous lysine residue is absent from FbpB-C and is instead replaced with a loop structure (*i.e.*, Q394 to Q405) that interrupts the fifth alpha helix (20). In the FbpC-C crystal structure, the K2 site exhibits an unwinding of fifth alpha helix into a larger loop structure (residues 344–354), more similar to what was observed for this site in FbpB-C (20). However, unlike FbpB-C, and despite the noted differences in secondary structure, FbpC-C presents a surface-exposed lysine residue at a structurally homologous position to BBK32 K327 and FbpA K343 (Figs. 1, *B*–*D* and S2) (20, 31). Collectively, this analysis reveals that, while the core folds are similar, distinct structural features are concentrated at the K1 and K2 sites across the family of borrelial C1r inhibitors and in particular for *B. hermsii* FbpC.

### *B. hermsii* FbpC binds both active and zymogen forms of C1r and inhibits human complement

**Figure 2:**
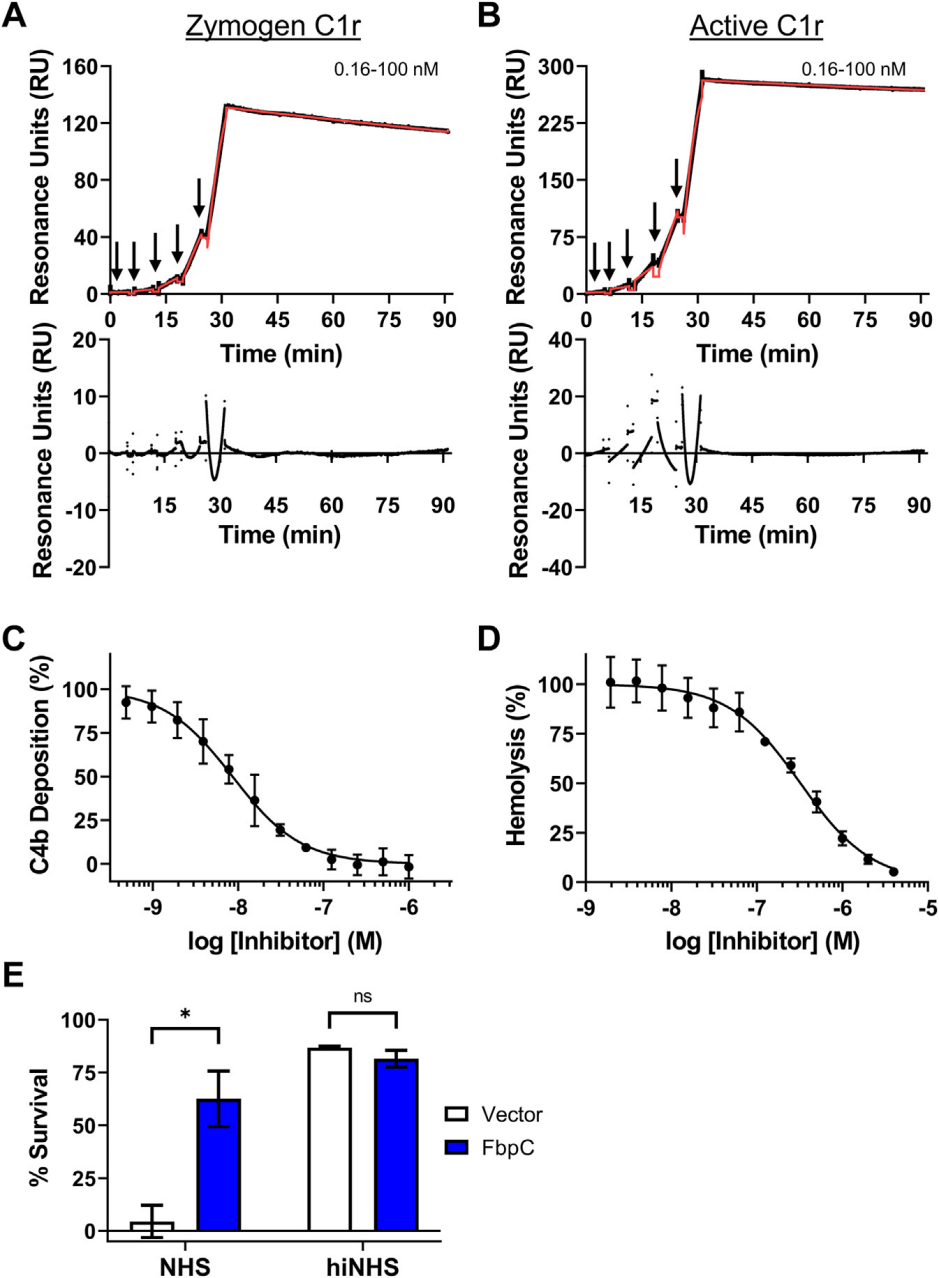
***Borrelia hermsii* FbpC C1r binding and complement activity assays.** Single-cycle SPR-binding assays using a 0.16 to 100 nM fivefold dilution series of (*A*) zymogen or (*B*) active C1r over FbpC-C. Sensorgrams are a representative injection series from three replicates with the raw sensorgrams drawn in *black* and kinetic fits traced in *red*. Calculated *K*_D_ values and association (*k*_a_) and dissociation (*k*_d_) rate constants for each analyte are shown in Table 2. The associated residual plots are shown below to provide information about the agreement of the fits to the raw data for each representative sensorgram. *C*, FbpC-C dose dependently inhibits C4b deposition in a CP-specific complement ELISA. ELISAs were performed in triplicate followed by a nonlinear variable slope regression fit to determine IC_50_ values present in Table 2. *D*, FbpC-C inhibits CP-mediated hemolysis in a dose-dependent manner with the associated IC_50_ value determined using nonlinear variable slope regression (Table 2). *E*, serum sensitivity assay demonstrating that *Borrelia burgdorferi* B314 ectopically expressing *B. hermsii fbpC* significantly protects against complement-mediated bacteriolysis when compared to a vector-only strain. Heat inactivated normal human serum (hiNHS) was used as a control. Statistical significance was determined using a two-way ANOVA and defined as *p* < 0.05. Error bars denote SD. ∗*p* < 0.05). CP, classical pathway; SPR, surface plasmon resonance.

Site-directed mutants and naturally occurring sequence variations at the K1 and K2 sites of BBK32 and Fbps alter their C1r binding and complement inhibitor activities (20, 30, 31). Considering this, and given the structural differences noted above, we next tested the C1r-binding properties of FbpC-C. Previously, we have shown that BBK32-C and FbpA-C bind with high affinity to both zymogen and active forms of human C1r, while FbpB-C is selective for the activated form (20, 30, 31, 32). To determine if FbpC-C binds zymogen and/or active human C1r, we generated a biosensor by immobilizing FbpC-C on a surface plasmon resonance (SPR) sensor chip and analyzed its binding to purified human zymogen C1r or activated C1r (Fig. 2, *A* and *B*) (20). Like BBK32-C and FbpA-C, FbpC-C interacted with both zymogen and active forms of purified human C1r with high affinity (*K*_D_ values of 1.2 and 0.3 nM, respectively) (Fig. 2, *A* and *B* and Table 2).

**Table 2:** C1r binding and CP inhibition data

Proteins	FbpC-C	FbpC-C R248A	FbpC-C H252A
SPR: Zymogen C1r *K*_D_ (nM)	1.2 ± 0.034	13 ± 6.6	N.D.
*k*_a_ (1/Ms)	4.4 × 10^4^ ± 1.2 × 10^4^	6.9 × 10^4^ ± 7.0 × 10^4^	N.D.
*k*_d_ (1/s)	5.4 × 10^−5^ ± 1.4 × 10^−5^	6.3 × 10^−4^ ± 3.6 × 10^−4^	N.D.
SPR: Active C1r *K*_D_ (nM)	0.3 ± 0.014	0.19 ± 0.048	2.4 ± 0.12
*k*_a_ (1/Ms)	4.8 × 10^4^ ± 0.048 × 10^4^	4.5 × 10^4^ ± 0.52 × 10^4^	5.1 × 10^4^ ± 0.63 × 10^4^
*k*_d_ (1/s)	1.4 × 10^−5^ ± 0.076 × 10^−5^	9.4 × 10^−6^ ± 3.1 × 10^−6^	1.2 × 10^−4^ ± 0.19 × 10^−4^
CP ELISA IC_50_ (nM)	8.6 (7.5–9.8)	23 (20–26)	300 (240–400)
CP Hemolysis IC_50_ (nM)	320 (270–380)	1200 (1100–1400)	N.D.

N.D. stands for not determined. SPR-binding data shown as average ± SD. IC_50_ values shown with corresponding 95% confidence intervals.

Next, we determined if FbpC-C inhibits the progression of the human CP. Consistent with the observed high affinity binding to C1r (Fig. 2), FbpC-C inhibited the human CP in a dose-dependent manner with an IC_50_ of 8.6 nM in an ELISA-based assay (Fig. 2*C* and Table 2). FbpC-C also protected antibody-opsonized sheep red blood cells from CP-specific hemolysis in a dose-dependent manner (Fig. 2*D*). We note that the weaker IC_50_ of 320 nM observed in this assay compared to the more sensitive C4b deposition ELISA assay is consistent with our previous observations of BBK32-C inhibitory activity (20, 30). We then asked if ectopic expression of full-length FbpC on a surrogate borrelial strain could prevent complement-mediated bacteriolysis. To this end, we used a high-passage *B. burgdorferi* strain (*B. burgdorferi* B314) that lacks all linear plasmids and is highly sensitive to human serum-based complement-mediated killing (20, 32, 37). *B. burgdorferi* B314 producing full-length, surface-expressed FbpC (Fig. S3) showed a significant increase in resistance to human serum killing when compared to a *B. burgdorferi* B314 vector-only control (Fig. 2*E*), indicating that FbpC is sufficient to protect this strain from human complement-mediated bacteriolysis. FbpC-C also was unable to inhibit the lectin pathway of complement at concentrations up to 1 μM, indicating that FbpC-C is CP-specific like BBK32-C (Fig. S4) (30). Taken together, these results show that *B. hermsii* FbpC binds to human C1r, inhibits the CP, and protects the serum-sensitive *B. burgdorferi* surrogate strain B314 from complement-mediated killing.

### MD simulations of borrelial C1r inhibitor proteins

With crystal structures of a representative family member of each borrelial C1r inhibitor in hand (PDB: 6N1L, 7RPR, 7RPS, 8EC3), we next set out to utilize MD simulations to test the hypothesis that BBK32 and/or Fbps are structurally dynamic at either the K1 site, K2 site, or both functional sites. This hypothesis was primarily formed from the crystallographic studies showing an increase in temperature factors in the K1 sites of all four proteins (Fig. S5*A*), a lack of K1 site electron density for FbpC-C, and a lack of K2 site electron density for FbpB-C (20). Each of these observations suggested increased dynamics at functionally critical regions of borrelial C1r inhibitor proteins. MD simulations predict how atoms in a protein move over time (38) and have become a powerful approach for understanding protein motions that occur on the ns-μs time scale, which include conformational changes of side chains and loops (38, 39, 40, 41). To study these motions in borrelial C1r inhibitors, we carried out 1 μs MD simulations on *B. burgdorferi* BBK32-C, *B. miyamotoi* FbpA-C and FbpB-C, and *B. hermsii* FbpC-C in triplicate. Representative simulations for each inhibitor are shown in Supplemental Movies 1–4.

### Initial analysis of MD simulations

**Figure 3:**
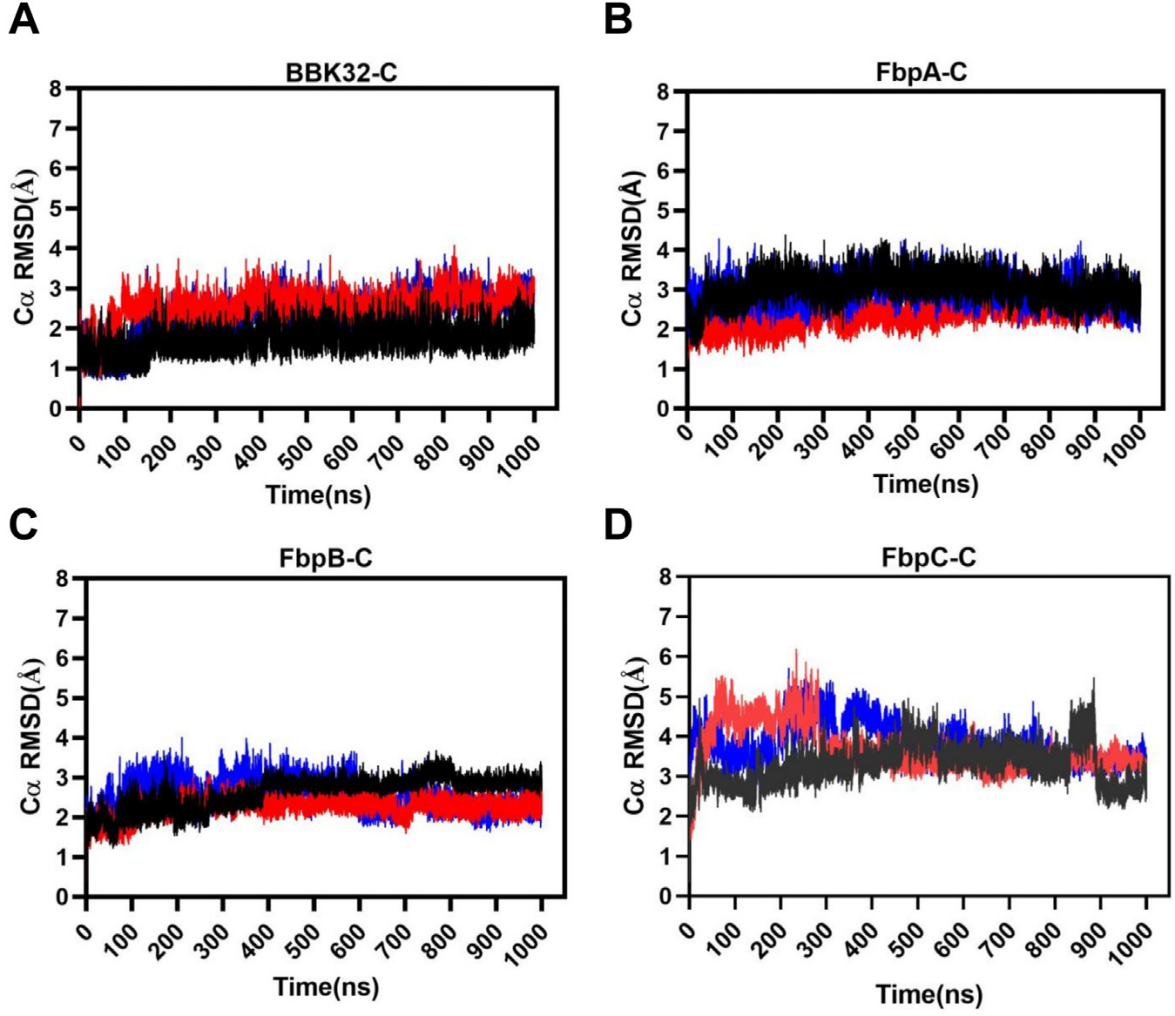
**Cα RMSD plots.** The Cα RMSD was tracked over the course of each 1 μs simulation for (*A*) BBK32-C, (*B*) FbpA-C, (*C*) FbpB-C, and (*D*) FbpC-C. Simulation replicate 1 (*black*), 2 (*blue*), and 3 (*red*) are shown.

RMSD describes the average displacement of atoms at a snapshot in time relative to a reference frame (*i.e.*, the crystal structure) and is a measure of the stability of a given simulation (42). Thus, RMSD reports on global conformational changes of protein structure throughout the simulation. RMSD values of the alpha carbons (Cα RMSD) of BBK32-C, FbpA-C, FbpB-C, and FbpC-C showed that most simulations converged after 100 to 200 ns, with FbpC-C simulations converging near 500 ns (Fig. 3, *A*–*D*). BBK32-C demonstrated the lowest RMSD values, ranging across the three simulations at 1.70 to 2.52 Å, followed by FbpA-C (2.36–3.04 Å) and FbpB-C (2.25–2.60 Å). FbpC-C RMSD values were relatively higher over the other three inhibitors, ranging between 3.29 and 3.81 Å, suggesting an increase in overall structural flexibility of FbpC-C compared to BBK32-C, FbpA-C, and FbpB-C.

**Figure 4:**
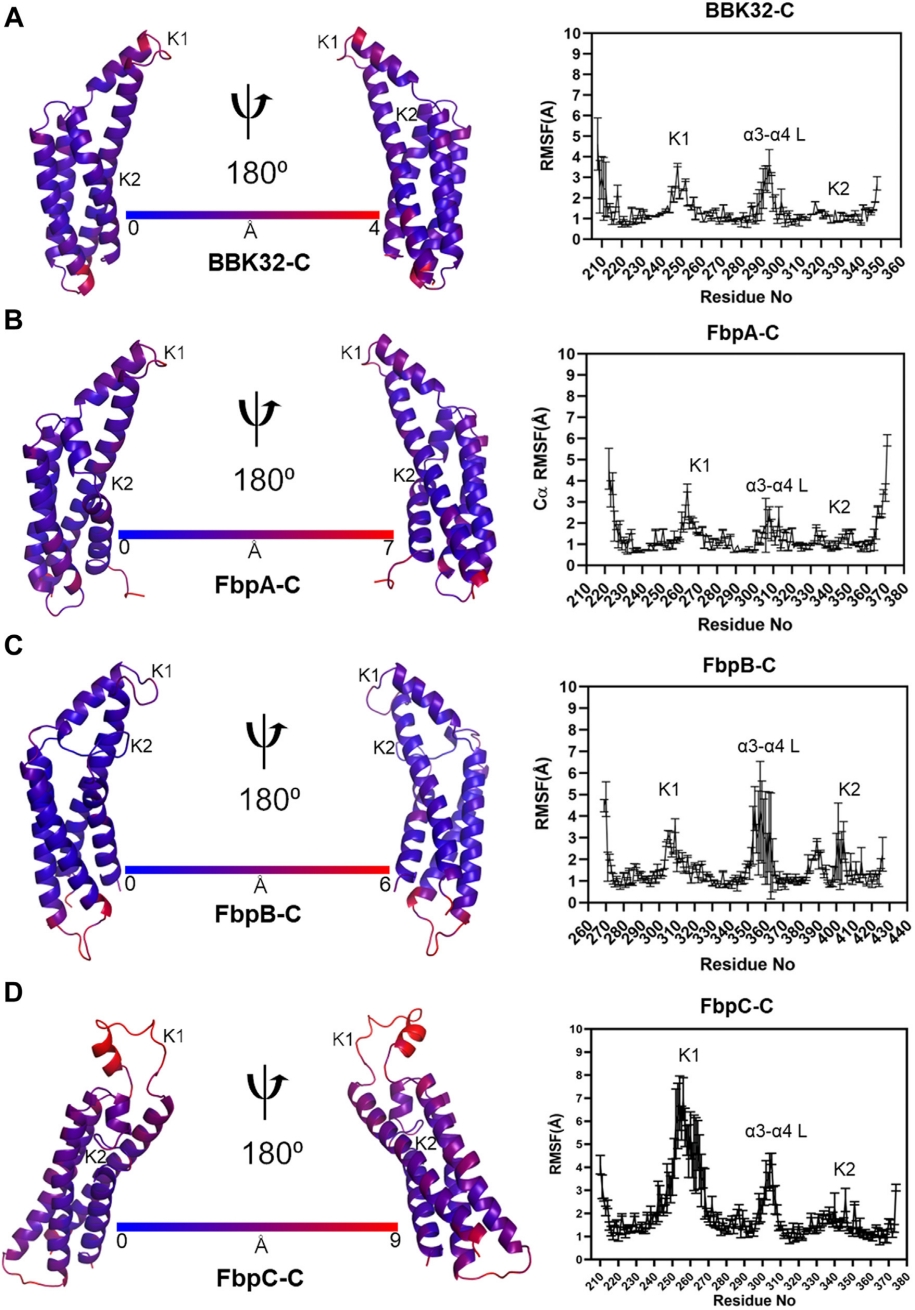
**RMSF measurements.** Residue root mean square fluctuation (RMSF) values from a representative MD simulation of (*A*) BBK32-C, (*B*) FbpA-C, (*C*) FbpB-C, and (*D*) FbpC-C were mapped onto structural models of each protein. *Dark blue* indicates the least flexible residues, while *dark red* indicates the most flexible residues within each protein. On the *right panels*, RMSF values are shown as an average and SD of three replicate simulations for each residue. The location of the α3-α4 L, K1, and K2 sites are marked. MD, molecular dynamics.

Next, we evaluated local residue flexibility by calculating root mean square fluctuations (RMSFs). RMSF is conventionally calculated for groups of atoms that comprise a residue and is compared to the initial starting structure (43). Residue RMSF measures can identify amino acids that contribute most to the molecular motions of a protein in the timeframe of the simulation. As judged by residue RMSF values (Fig. 4), the K1 site residues form one of the most dynamic regions within each of the borrelial C1r inhibitor proteins. In all simulations, common areas of high residue RMSF values are found at the N- and C-terminal residues of each protein and on the loop that connects alpha helices 3 and 4 (Fig. 4). Taken together, analysis of 1 μs MD simulations of BBK32-C, FbpA-C, FbpB-C, and FbpC-C suggests that a major functional site (*i.e.*, the K1 site) is dynamic in all four inhibitors with relatively increased dynamics observed for FbpC-C. While relatively less flexible in all proteins compared to the K1 site, the K2 site in FbpB-C is more dynamic than BBK32-C, FbpA-C, and FbpC-C, which is consistent with the lack of electron density for this loop in the prior FbpB-C crystal structure (20).

### K1 and K2 sites of borrelial C1r inhibitors display anticorrelated motions

To further investigate the dynamics of the K1 and K2 sites in borrelial C1r inhibitors, we carried out all atom normal mode analysis (Fig. S5*B*) (44, 45). Normal mode analysis has been used to detect larger amplitude motions in proteins which are involved in biological functions (46, 47, 48, 49, 50, 51, 52). Separate all atom normal modes for BBK32-C, FbpA-C, FbpB-C, and FbpC-C showed that the largest amplitude motions localize to the K1 site with other small amplitude normal modes distributed throughout each of the proteins. Interestingly, normal modes at the K2 sites in all proteins are significantly less in amplitude but are directed opposite to that of the corresponding K1 site (Fig. S5*B*).

**Figure 5:**
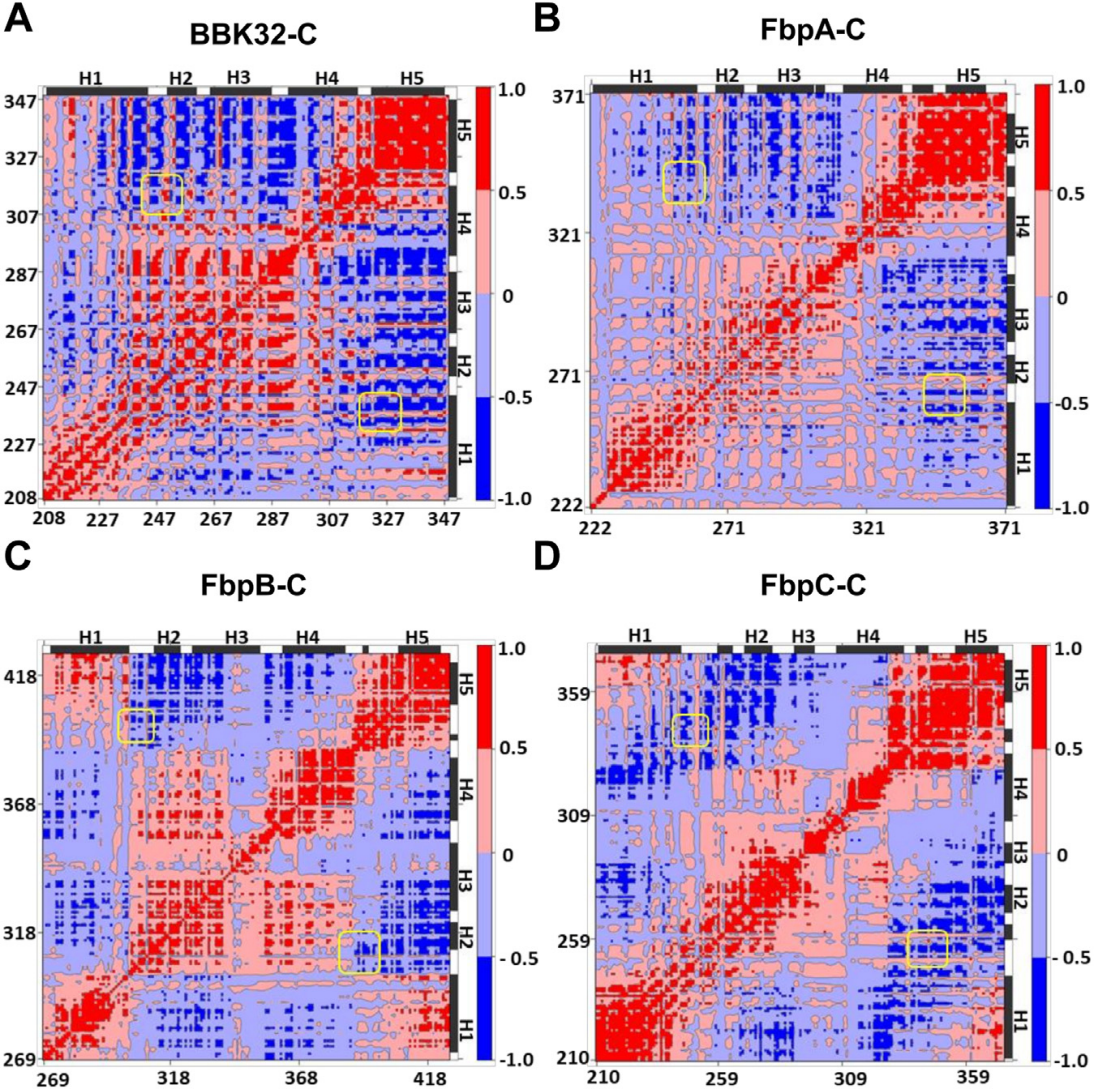
**Cα DCCM maps for borrelial C1r inhibitors.** Dynamic cross-correlation matrices (DCCMs) maps are shown for representative simulations of (*A*) BBK32-C, (*B*) FbpA-C, (*C*) FbpB-C, and (*D*) FbpC-C. Positive values (*red*) represent correlated motions, while negative values (*blue*) represent anticorrelated motions. Regions of the maps corresponding to K1 and K2 sites are highlighted by *yellow boxes*. Residues involved in helical secondary structure are denoted by *black bars* where H1-H5 denote the positions of alpha helices 1 to 5 relative to the BBK32-C structure.

To further investigate the relative conformations of the K1 and K2 sites in these proteins, we carried out dynamic cross correlation matrix (DCCM) analysis of the MD simulations. DCCM measures positional displacements of the Cα atoms at a sample rate of 100 ps, which provides a graphical representation of time-correlated motions of residues within a given protein (53, 54, 55, 56). Correlated motions (Fig. 5, red) are apparent for the helices that form the core of each borrelial C1r inhibitor. Strikingly, in each protein, the residues that comprise the K1 and K2 sites (yellow boxes) move in an anticorrelated manner relative to one another (Fig. 5, blue). This analysis is consistent with the results of the normal mode analysis (Fig. S5*B*) and, taken together, suggests that borrelial C1r inhibitor proteins may utilize a highly flexible K1 site and anticorrelated motion at the K2 site to generate variation in the distance between the two functional sites.

### Conformational plasticity of the K1 and K2 sites in borrelial C1r inhibitors

Analysis of the MD simulations led to a new hypothesis that the two major C1r-binding surfaces on free borrelial C1r inhibitors (*i.e.*, K1 and K2 sites)—which are separated in space by ∼25 to 30 Å—are sampling a larger range of conformations in their unbound forms, particularly for FbpC-C. To test this, we first defined the distance between the K1 and K2 sites in each protein by measuring the distance between two representative residues from each site using the Cα atom (*i.e.*, main chain distance) or a distal atom from the side chain as follows: (i) BBK32-C; K1: R248, K2: K327; (ii) FbpA-C; K1: R264, K2: K343; and (iii) FbpB-C; K1: R309, K2: N402. As noted above, the FbpC sequence differs in this area from the other three proteins and a sequence alignment predicts that H252 is analogous to the K1 site arginine residues from BBK32-C, FbpA-C, and FbpB-C. However, we noted that an arginine residue is also present on the flexible K1 site loop in FbpC-C (*i.e.*, M_247_-RMSGHST_254_; note that H252 is also underlined here) (Fig. S2). Thus, we tracked two measurements for FbpC-C: (1) K1: R248; K2: K345 and (2) K1: H252; K2: K345.

**Figure 6:**
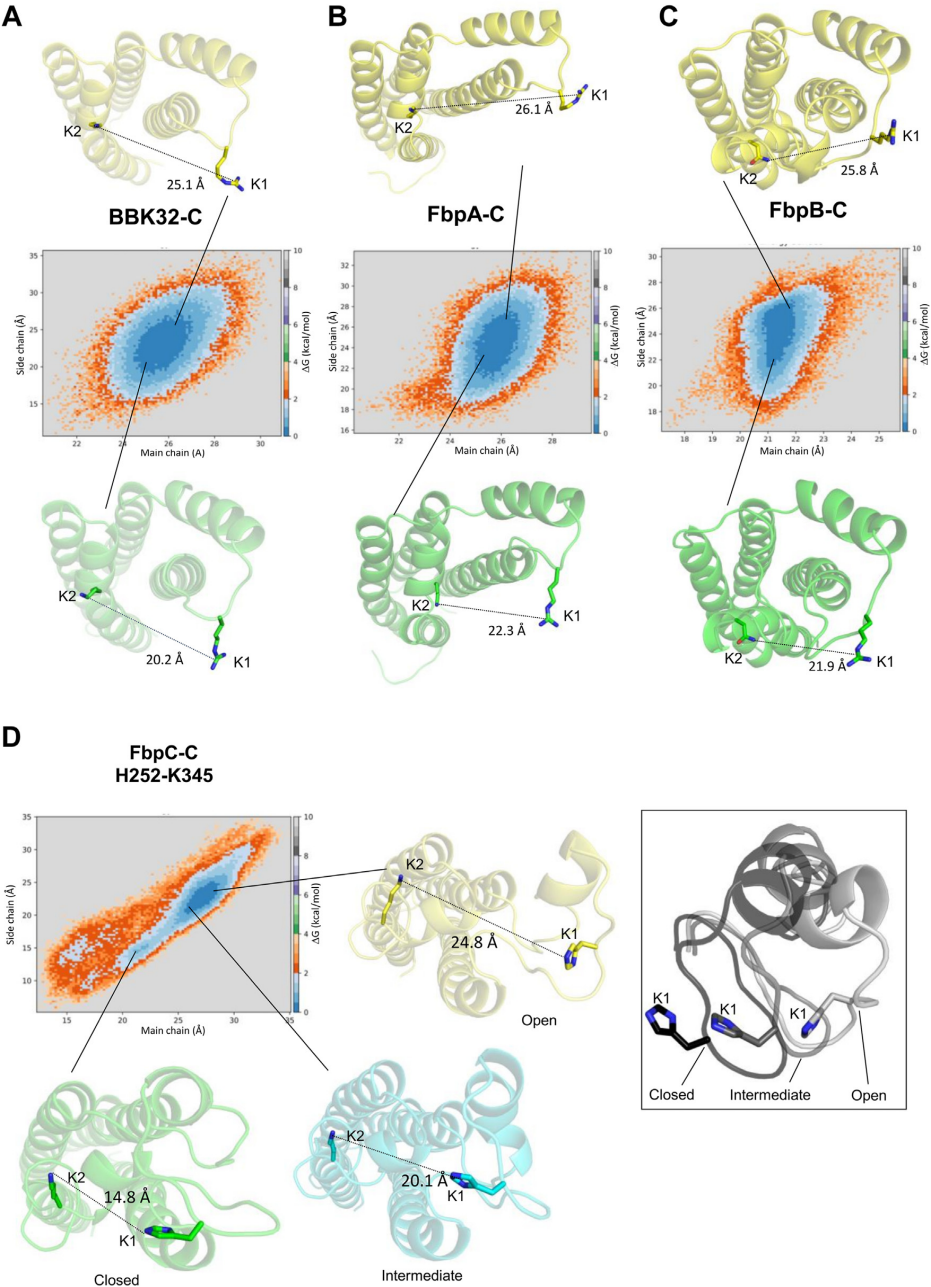
**Free energy surfaces of borrelial C1r inhibitors in relationship to the main chain and side chain distances between the K1 and K2 sites.** Two-dimensional free energy surfaces are shown for (*A*) BBK32-C, (*B*) FbpA-C, and (*C*) FbpB-C. Distance between the K1 and K2 sites at main chain and side chain atoms are used as order parameters. Main chain distances were measured using the Cα atom of each selected residue. The residues used for these measurements are defined as BBK32-C (K1: R248, K2: K327); FbpA-C (K1: R264, K2: K343); FbpB-C (K1: R309, K2: N402). Side chain distances are defined as distances between the K1 site Arg-CZ atom and K2 site Lys-NZ atom in BBK32-C and FbpA-C. In FbpB-C, side chain distances were defined as distances between K1 Arg-CZ and ND2 atom of N402. A relative energy scale is shown on the *right* y-axis with *dark blue* indicating the lowest energy conformations. Representative conformations from the edge of each energy minima are shown with K1 and K2 side chain distance measurements drawn. *D*, free energy surfaces generated for FbpC-C using the NE2 atom of H252 and the NZ atom of K345 to measure the side chain distance. Representative conformations are shown for three energy minima for the most closed (*green*), most open (*yellow*), and median distances (*cyan*). A close-up view of the K1 site and the associated secondary structure of open, median, and closed states are shown on the *right*.

Using these measures, we first analyzed how K1-K2 site distances change during the simulation (Fig. S6, *A*–*E*). Distances between K1 and K2 sites in BBK32-C ranged from 22 to 30 Å (main chain; average = 25.3 Å) and 12 to 35 Å (side chain; average = 23.3 Å). We then projected two-dimensional free energy surfaces using main chain and side chain K1-K2 site distances as order parameters (57, 58). This approach was taken to assess the relative K1-K2 site distances of energetically favorable conformations within unbound forms of each protein. This analysis suggests that BBK32-C samples energetically favorable conformations that place the K1 and K2 sites between main chain distances of 24 to 27 Å and side chain distances of 18 to 25 Å apart (Fig. 6*A*, dark blue). Similar results were obtained for FbpA-C with the most favorable conformations leading to K1-K2 distances between 25 to 27 Å (main chain) and 20 to 28 Å (side chain) (Fig. 6*B*). FbpB-C produces a similar shape of the free energy surface plot and indicates that the most favorable K1-K2 site distances range from 19 to 22 Å (main chain) and 22 to 26 Å (side chain) (Fig. 6*C*). These results reveal that in the unbound form, BBK32-C, FbpA-C, and FbpB-C sample a similar range of energetically favorable states that modulate the relative positions of the two major C1r-binding surfaces (Fig. 6, *A*–*C*), which presumably adopt a fixed distance in the bound state.

Unlike the other energy contours, FbpC-C exhibits three clear energy minima when R248 is used in the measurement of the K1-K2 site distance (Fig. S6*F*). The largest basin, represented by the cyan structure in Fig. S6*F*, involves energetically favorable conformations that produce K1-K2 site distances of 18 to 20 Å (main chain) and 18 to 23 Å (side chain). A more closed conformation (represented by the green structure) and more open conformation (represented by the yellow structure) are also apparent (Fig. S6*F*). A distinct free energy surface is also seen when H252 is used to measure the K1-K2 site distance in FbpC-C (Fig. 6*D*). Two energy minima are observed that collectively span 21 to 30 Å (main chain) and 14 to 25 Å (side chain). This suggests that FbpC-C adopts energetically favorable conformations that produce a wide range of distances of the K1 and K2 sites. Also, in contrast to the other three inhibitors, the secondary structure of the K1 site undergoes considerable rearrangement during the time course of the simulation. As an example, the conformation of the more closed state (Fig. 6*D*, green) involves an unwinding of alpha helix 2. Likewise, the helical structure of the K1 site observed in the more open state (Fig. 6*D*, yellow) differs from that found in the central energy basin (Fig. 6*D*, cyan).

### Dynamic K1 site residues are important for FbpC-C function

**Figure 7:**
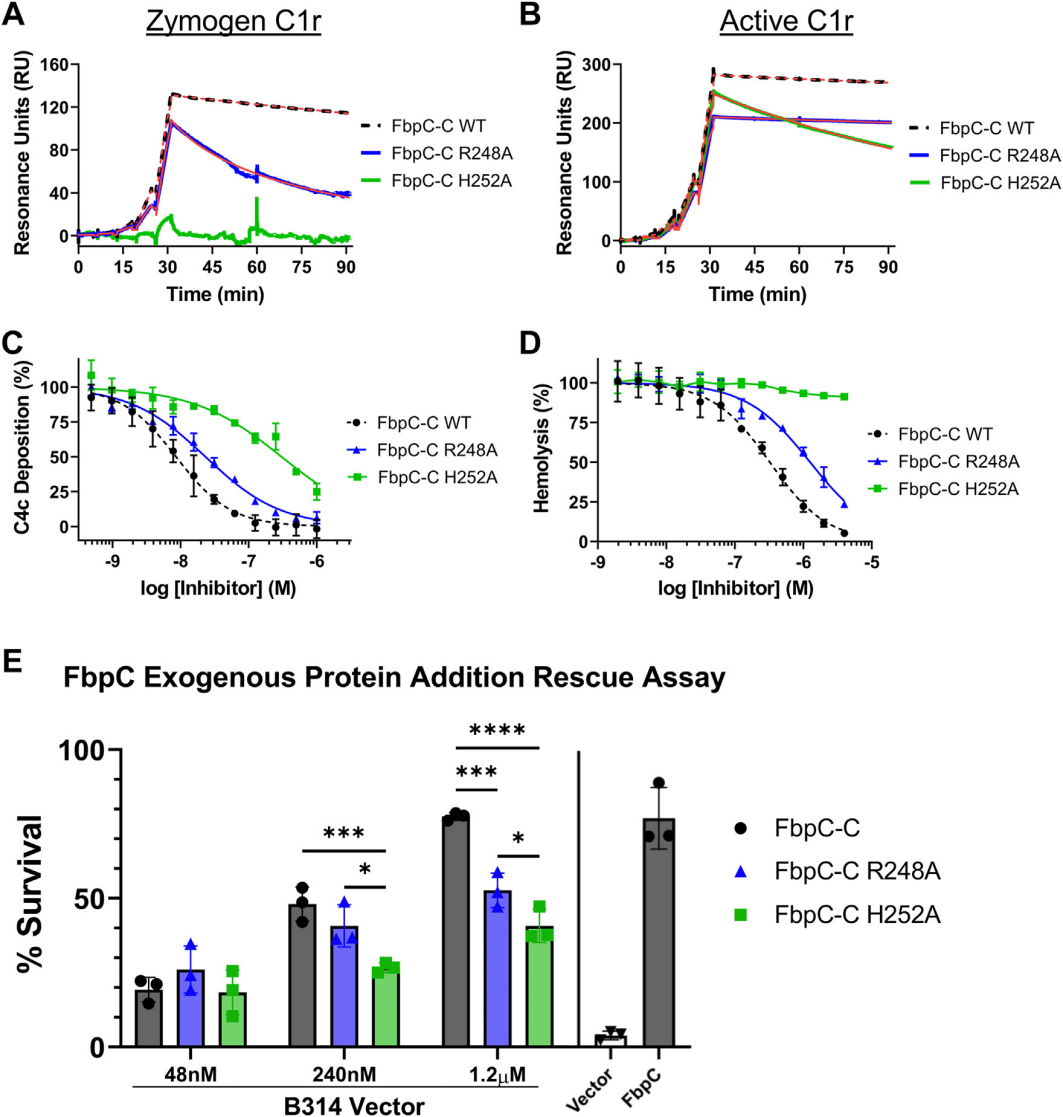
**Assessing the function of the K1 site residues of FbpC-C.** Single-cycle SPR-binding assays using a 0.16 to 100 nM five-fold dilution series of (*A*) zymogen or (*B*) active C1r over FbpC-C R248A and H252A. Sensorgrams are a representative injection series from three replicates with the raw sensorgrams overlaid with kinetic fits traced in *red*. A kinetic fit for zymogen C1r binding to FbpC-C H252A was unable to be determined and is shown only as the raw sensorgram. Calculated *K*_D_ values and association (*k*_a_) and dissociation (*k*_d_) rate constants for each analyte were determined and are shown as the mean ± SD (Table 2). Representative sensorgrams for analogous experiments using “wild-type” FbpC-C are replotted from Figure 2 for sake of comparison and are delineated by *dashed lines*. *C*, C4b deposition was dose dependently inhibited in a CP-specific complement ELISA incubated with FbpC-C R248A and FbpC-C H252A. CP ELISAs were performed in triplicate followed by a nonlinear variable slope regression fit to determine IC_50_ values (Table 2). Representative dose-response inhibition curves for analogous experiments using “wild-type” FbpC-C are replotted from Figure 2 for sake of comparison and are delineated by *dashed lines*. *D*, FbpC-C R248A was capable of dose dependently inhibiting CP-mediated erythrocyte hemolysis with the associated IC_50_ value determined using a nonlinear variable slope regression fit found in Table 2. FbpC-C H252A was unable to protect erythrocytes from CP-mediated cell lysis. Dose-response inhibition curves for hemolysis assays utilizing “wild-type” FbpC-C are replotted from Figure 2 for sake of comparison and are shown as *dashed lines*. *E*, the ability of soluble recombinant FbpC-C proteins to protect serum-sensitive *Borrelia burgdorferi* strain B314 was assessed. Purified exogenous recombinant FbpC-C, FbpC-C R248A, or FbpC-C H252A were added to final concentrations of 48 nM, 240 nM, or 1.2 μM, approximating to five-fold less than, equal to, and five-fold greater than the concentration of C1r in the human serum sample used. Survival was compared to that of B314 pBBE22*luc* alone (B314 Vector) and of B314 pAP20 (FbpC), which produces *Borrelia hermsii* FbpC, as described in Figure 2*E* for the serum-sensitivity assays. Error bars denote SD. ∗*p* < 0.05; ∗∗∗*p* < 0.0003; ∗∗∗∗*p* < 0.0001. CP, classical pathway; SPR, surface plasmon resonance.

There was ambiguity in determining which residue within the dynamic K1 site of FbpC-C (*i.e.*: R248 or H252) was involved in C1r interactions based on sequence and structural alignments (Figs. 1 and S2). Furthermore, the analysis conducted in Figures 6*D*, S5, and S6*F* suggest that both residues can adopt conformations that would feasibly allow them to interact with C1r, as judged by the previous studies with BBK32, FbpA, and FbpB (20, 32). To test if R248 and/or H252 contribute to C1r-binding affinity, we generated two site-directed alanine mutants, FbpC-R248A and FbpC-H252A, and assayed binding to C1r using SPR and in assays of complement activation (Fig. 7).

FbpC-R248A bound zymogen and active C1r with respective *K*_D_’s of 13 and 0.19 nM and dose dependently inhibited the CP with an IC_50_ of 23 nM, an overall 2- to 3-fold difference in inhibition. In contrast, FbpC-C H252A exhibited a weaker affinity for active C1r (*K*_D_ = 2.4 nM) than WT FbpC-C (*K*_D_ = 0.3 nM) and even more so for zymogen C1r where a *K*_D_ could not be reliably determined (Fig. 7, *A* and *B*). Consistent with this C1r-binding deficit, CP-specific ELISAs showed that the H252A mutation had a pronounced effect on CP inhibition with an IC_50_ value of 300 nM, compared to 8.6 nM for WT FbpC-C (Fig. 2*C* and replotted in Fig. 7*C* for comparison) (Fig. 7*C* and Table 2). Similarly, a deficit in the CP-specific complement-inhibitory activity of FbpC-C R248A was measured in hemolysis assays, while more dramatically, FbpC-C H252A did not significantly inhibit in this assay format at concentrations up to 4 μM (Fig. 7*D*). We also asked what the importance of each the K1 site residues was for FbpC-C’s activity in a functional bacterial cell-based assay (20, 30). Using the serum-sensitive *B. burgdorferi* B314 pBBE2*2luc* background coupled with normal human serum (NHS), we exogenously added in each recombinant protein: FbpC-C WT, FbpC-R248A, or FbpC-H252A. While FbpC-R248A exhibited a reduction in its ability to protect *B. burgdorferi* B314 from serum-mediated killing relative to its WT counterpart, FbpC-H252A was even further impaired in its ability to prevent killing (Fig. 7*E*). Both strains were compared to *B. burgdorferi* B314 expressing FbpC (pAP20) as a positive control and vector-only B314 pBBE22*luc* as a negative control for complement resistance. These data highlight that both residues affect binding and inhibition of C1r to various degrees; however, mutation of H252 had the largest effect. Taken together, these results underscore the importance that dynamic residues of the K1 site play in the overall complement-inhibitory properties of FbpC-C.

## Discussion

To colonize and persist following infection, many bacterial pathogens produce outer surface–associated proteins that specifically interact with host proteins. These bacteria-host protein–protein interactions have diverse functions, including facilitating pathogen adherence to tissues, promoting invasion of cells, and providing protection against host immune defenses (1, 2, 3, 4, 8, 17, 22, 59, 60, 61). Lyme disease spirochetes produce at least 80 outer surface lipoproteins, many of which are upregulated upon transmission from the vector to the host (62). While the functions of most of these proteins remain unknown, nearly a dozen have been shown to bind directly to protein components of the innate immune system known as the complement cascade (3, 9). Some of these complement evasion lipoproteins act as highly specific inhibitors of complement proteases, including a family of C1r inhibitors prototyped by *B. burgdorferi* BBK32 and the orthologous Fbp proteins of relapsing fever and *B. miyamotoi* spirochetes (18, 20, 30, 31, 32, 33). In this study we begin to assess how sequence diversity across C1r borrelial inhibitors influences their structure and function.

Our previous investigation into the molecular basis for C1r inhibition by BBK32, FbpA, and FbpB defined two C1r-interacting surfaces located on the alpha helical C-terminal region of each protein referred to herein as K1 and K2 sites. However, the structure and C1r inhibitory activity of a representative FbpC protein had not been determined. Interestingly, the crystal structure of *B. hermsii* FbpC-C, presented here, exhibited structural deviations at both the K1 and K2 sites compared to BBK32-C, FbpA-C, and FbpB-C (Fig. 1). In particular, the conformation of the K1 site involved a repositioning of alpha helix 2 coupled with the absence of strong electron density for eight residues that connect alpha helix 1 and alpha helix 2, which suggested this region was highly flexible (Fig. 1*A*). We cannot rule out the influence of the crystallization process/conditions in stabilizing the conformation observed for the FbpC-C K1 site, but we note that BBK32-C, FbpA-C, and FbpB-C were all crystallized such that the K1 sites were in a more closed conformation. Despite the structural differences for FbpC-C, we showed that it retains high affinity for human C1r and inhibits human complement in a manner similar to BBK32 and *B. miyamotoi* FbpA (Fig. 2).

Our structure-based knowledge of bacteria-host protein–protein interactions is often derived from static three-dimensional models produced by X-ray crystallography. Powerful new machine learning–based approaches, such as AlphaFold2, also produce static representations of protein structures. However, proteins are dynamic entities, and their native conformational variations can dramatically influence their functions. Crystallographic B-factors—or the noted correlation of pLDDT values to protein order in AlphaFold2 models (Figs. S1 and S5*A*) (63)—provides some information on protein motions. However, their utility is somewhat limited as protein dynamics occur across different timescales and are associated with different distances. Here, we studied protein dynamics of C1r borrelial inhibitors using MD simulations, which allows for visualization of protein dynamics on the ns-μs time scale (38, 40). This enabled us to observe motions ranging from side chain fluctuations to larger conformational changes associated with loop movements and secondary structure changes, particularly at the K1 site (Figs. 3 and 4) (39, 40). Previous studies using MD have discovered novel functional domains of proteins (64) and aided in understanding how surface receptors transmit their signals within the cell (65). Regarding bacterial proteins, MD simulations have been used to reveal how polymorphisms in FnBPA of *Staphylococcus aureus* increase affinity for human fibronectin, resulting in an increased ability to colonize medical implant devices (66). To our knowledge, the application of MD for borrelial proteins has been limited to studying DNA–protein interactions or periplasmic proteins (67, 68) and is thus a novel approach for the study of borrelial outer surface lipoproteins.

The crystal structure of FbpC-C (Fig. 1) and results from RMSF analysis (Fig. 4) for the MD simulations suggested that the K1 sites of borrelial C1r inhibitors were highly dynamic. Interestingly, DCCM and normal mode analyses also indicated that the K1 and K2 site of each protein moved in an anticorrelated fashion (Figs. 5 and S5*B*). Free energy surface analysis showed that BBK32 and Fbp proteins adopt energetically favorable “open” and “closed” states defined by the variation in distance between the functional K1 and K2 sites (Figs. 6 and S6). FbpC-C was particularly dynamic at the K1 site, and our investigations into the flexible loop of the K1 site of FbpC-C by site-directed mutagenesis showed that the H252A had decreased CP inhibition in three different assays and decreased binding to C1r (Fig. 7). In contrast, the R248A mutant protein exhibited a smaller decrease in inhibition of the CP (Fig. 7). These data, in combination with previous mutants of the K1 site of BBK32-C and FbpA-C (20, 31), demonstrate that residues in dynamic regions of borrelial C1r inhibitors contribute significantly to their inhibitory functions.

We speculate that dynamics of the K1 and/or K2 sites may contribute to the selectivity of borrelial C1r inhibitors for specific conformations of the protease (*i.e.*, zymogen *versus* active) as has been reported for FbpB (20). However, additional study is required to fully understand this relationship that includes consideration of protein dynamics of the proteases themselves, particularly within the context of the larger C1 complex (*i.e.*, C1qC1r_2_C1s_2_). While we have shown here that FbpC does not inhibit a closely related complement pathway (*i.e.*, LP; Fig. S4), it is possible that the sequence diversity across BBK32/Fbp proteins may generate proteins that are capable of inhibiting more distantly related host serine proteases. Furthermore, complement proteins are highly conserved across vertebrates, including for C1r. However, we have previously noted that differences within vertebrate C1r-SP are concentrated in loop structures such as the B-loop. We hypothesize that borrelial pathogens, which naturally encounter disparate vertebrate reservoirs, may have evolved borrelial C1r inhibitors that are evolutionarily optimized for particular C1r sequences. While this possibility remains to be formally tested, evidence for this type of molecular host adaptation has been recently demonstrated for a different borrelial anticomplement lipoprotein that can bind human factor H, notably CspZ (69, 70, 71). Future work in this area should consider how protein dynamics contributes to host adaption for vector-borne pathogens like Lyme disease and relapsing fever spirochetes.

In summary, we characterized the structure and function of the C1r-binding domain of *B. hermsii* FbpC and carried out a detailed MD simulation study for the family of borrelial C1r inhibitors. This investigation reveals an unexpected plasticity in the structures of these proteins and uncovers the existence of dynamic motions within key functional sites. These observations improve our understanding of how outer surface bacterial proteins modulate the host immune response and provide novel insight into how protein dynamics may influence their functions.

## Experimental procedures

### Plasmid constructs

The following constructs for *B. hermsii* HS1 *fbpC* (GenBank #: ADN26265.2) were subcloned into pT7HMT (72). *Escherichia coli* codon-optimized DNA fragments that encode for the C-terminal complement-inhibitory domain of FbpC, as well as site-directed mutants (FbpC-C, FbpC-C R248A, and FbpC-C H252A) corresponding to residues 212 to 374, were obtained from IDT Technologies gBlock Gene Fragment Service. Residue numbering is based on UniProt numbering (G9BXS5). Each FbpC-C DNA fragment was subcloned into pT7HMT as previously described (20, 30, 31, 32).

The *B. burgdorferi* expression construct for *B. hermsii fbpC* was assembled using the NEBuilderHiFI DNA Assembly Cloning Kit as described (20, 32). Briefly, the *fbpC* gene was PCR-amplified using primers BhFbpCUSF and BhFbpCDSR (Table S1) from *B. hermsii* strain HS1 genomic DNA using parameters as previously indicated (20, 32). The *bbk32* promoter was amplified using primers K32PromoterUSF and K32PBhFbpCFusR (Table S1). Following assembly and confirmation by dideoxy sequencing (not shown), the entire construct was amplified and cloned into pBBE22*luc* as previously outlined (20, 32). Transcriptional linkage of the *B. hermsii fbpC* gene with the *bbk32* promoter from *B. burgdorferi* ensured transcription of *fbpC* in B314. The resulting plasmid, which encodes the P_*bbk32*_-*fbpC* construct, was designated pAP2.

### Transformation of *B. burgdorferi*

*B. burgdorferi* strains used in this study are shown in Table S2. Transformation of strain *B. burgdorferi* B314 with the plasmid construct pAP2 was performed as previously described (31, 73). The presence of plasmid pAP2 was selected in complete BSK-II media using kanamycin at a final concentration of 300 μg/ml.

### Immunoblots

Western immunoblots were conducted to detect FbpC and BBK32 from constructs produced in *B. burgdorferi* strain B314. Protein lysates were resolved by 12.5% SDS-PAGE and transferred to polyvinylidene difluoride membranes. Following blocking, membranes were incubated with either a polyclonal mouse antibody to *B. hermsii* FbpC (diluted 1:5000), a monoclonal antibody to BBK32 (diluted at 1:20,000), or *B. burgdorferi* strain B31 FlaB (diluted at 1:10,000; Affinity BioReagents), washed, and then incubated with a 1:10,000 dilution of goat anti-mouse IgG-HRP (Thermo Fisher Scientific). The blots were processed to visualize the immune complexes as previously described (31).

### Proteinase K accessibility assays

Surface expression of *B. hermsii* FbpC in strain B314 was determined using proteinase K accessibility assays as described (20) with a polyclonal antibody against FbpC-C. The specificity of the polyclonal FbpC antibody was tested against strains B314-pBBE22*luc* (vector control) and B314-pCD100 (produces BBK32). A monoclonal antibody to BBK32 was used to confirm that *bbk32* was not being expressed and to test whether this BBK32-specific monoclonal cross reacted with FbpC when produced in B314-pAP2.

### Protein production and purification

Recombinant FbpC-C, FbpC-C R248A, and FbpC-C H252A proteins were expressed and purified as in (20). Briefly, after elution of the Ni column using the elution buffer (20 mM Tris (pH 8.0), 500 mM NaCl, 500 mM imidazole), the proteins were exchanged into 20 mM Tris (pH 8.0), 500 mM NaCl, 10 mM Imidazole using a Desalting 26/10 column (GE Healthcare). The His-tag was then removed by incubation with the tobacco etch virus protease and 5 mM β-mercaptoethanol overnight at room temperature. The tobacco etch virus–digested proteins were separated from the cleaved His-tag product on an AKTA Pure 25L FPLC using a 5 ml HisTrap-FF with the captured flowthrough further purified using a HiLoad Superdex 75 PG gel filtration column (GE Healthcare). A monodisperse peak was obtained and assessed for purity using SDS-PAGE gel analysis, then pooled, concentrated, and exchanged into HBS buffer (10 mM Hepes [pH 7.3], 140 mM NaCl). Purified full-length zymogen and active forms of C1r were obtained from Complement Technology, Inc. Multiple sequence alignments were generated using Clustal Omega (74).

### Generation of polyclonal antibodies against *B. hermsii* FbpC

Polyclonal antibodies reactive with *B. hermsii* FbpC were generated by immunizing female C57BL/6 mice intradermally with 25 μg of purified FbpC-C in an equal volume of PBS and TiterMax Gold Adjuvant (Sigma Aldrich) as outlined (75). Two weeks after the initial immunization, mice were boosted with 25 μg of FbpC-C. Two weeks after boosting, mice were euthanized and blood was immediately isolated by exsanguination, allowed to clot, and serum removed after low-speed centrifugation. Reactivity and specificity of the serum sample to *B. hermsii* FbpC was evaluated by Western Blot with B314-pAP2 relative to the vector-only control B314-pBBE22*luc*. All animal work was reviewed and approved by the Texas A&M University Institutional Animal Care and Use Committee (protocol numbers 2019-0422 and 2022-0309).

### Crystallization, structure determination, refinement, and analysis

FbpC-C_212–374_ was concentrated to 8 mg/ml in 10 mM Tris (pH 8.0), 50 mM NaCl buffer. Crystals of FbpC-C were obtained by vapor diffusion of sitting drops at 25 °C. Crystals grew in a condition containing 0.2 M MgCl_2_, 0.1 M Bis-Tris (pH 5.5), 25% PEG 3,350, and generally appeared in 3 days. Crystals were harvested and cryoprotected by supplementing the crystallization buffer with 10% glycerol.

Monochromatic X-ray diffraction data were collected at 1.0 Å wavelength using beamline BM-22 of the Advanced Photon Source (Argonne National Laboratory). Diffraction data were integrated, scaled, and reduced using the HKL2000 software suite (76) and assessed for data quality (77) (Table 1). FbpC-C crystals grew in space group P 2_1_ and contained a single copy within the asymmetric unit. Initial phases for FbpC-C were obtained by molecular replacement using the predicted FbpC-C structure from AlphaFold2 (Fig. S1) (35). Following an initial round of refinement, the model for FbpC-C was completed by a combination of automated chain tracing using PHENIX.AUTOBUILD (76, 78, 79) and manual building using COOT (80). The final model was completed upon iterative cycles of manual rebuilding and refinement using PHENIX.REFINE (76, 78, 79). Residues 247 to 254 and 301 to 304 were not modeled in the final refined structure due to poor electron density. Refined coordinates and structure factors have been deposited in the Protein Data Bank, Research Collaboratory for Structural Bioinformatics, Rutgers University (www.rcsb.org/) under the PDB ID 8EC3. A description of crystal cell constants, diffraction data quality, and properties of the final refined model can be found in Table 1. Representations of the protein structures and electron density maps were generated by PyMol (www.pymol.org/).

### AlphaFold2 modeling

AlphaFold2 was used to predict the protein structure for *B. hermsii* FbpC_212–374_ (35) using an AlphaFold2 Colab implementation (81) and subjected to default Amber relaxation. The corresponding pLDDT values were used to evaluate residue-level confidence in the predicted structure (35).

### Surface plasmon resonance

SPR was performed on a Biacore T200 instrument at 25 °C using a flowrate of 30 μl/min. HBS-T running buffer was used (10 mM Hepes (pH 7.3), 140 mM NaCl, and 0.005% Tween 20), which was supplemented with 5 mM CaCl_2_. FbpC-C was immobilized on a CMD200 sensor chip (Xantec Bioanalytics) *via* standard amine coupling as before (20, 30, 31, 32). The immobilization density for FbpC-C was 1138.2 RU, FbpC-C R248A was 975.2 RU, and FbpC-C H252A was 1082 RU. Zymogen or active C1r-binding data was obtained using a single cycle injection strategy (20) whereby a five-fold dilution series of either C1r zymogen or active C1r ranging from 0 to 100 nM was injected over the immobilized FbpC-C biosensor. Data was obtained in triplicate with each cycle having association times of 5 min and a final dissociation time of 1 h. Surfaces were regenerated to baseline with two 1 min injections with regeneration buffer (0.1 M glycine [pH 2.2], 2.5 M NaCl). Equilibrium dissociation constants (*K*_D_) and kinetic rates were obtained from the resulting kinetic fits obtained using Biacore T200 Evaluation Software (GE Healthcare; https://www.cytivalifesciences.com/en/us).

### ELISA-based complement-inhibition assay

ELISA-based assays that utilized IgM as a specific activator of the CP or mannan as an activator for the lectin pathway as described in (20, 30, 31, 32, 82) were used to assess complement inhibition by FbpC-C or the site-directed FbpC-C mutants. Briefly, FbpC-C, FbpC-C R248A, or FbpC-C H252A were serially diluted (two-fold dilution range: 1000 nM to 0.5 nM) and incubated with 2% NHS (Innovative Research) for CP-specific ELISAs. ELISAs specific for the lectin pathway contained single 1 μM concentrations of FbpC-C or BBK32-C incubated with 2% C1q-depleted NHS (Complement Technologies). Serum incubations were performed in complement ELISA buffer (10 mM Hepes pH 7.3, 140 mM NaCl, 2 mM CaCl_2_, 0.5 mM MgCl_2_, and 0.1% gelatin). A primary mouse antibody α-C4c (Quidel) was diluted to 1:10,000 followed by a secondary goat α-mouse antibody conjugated to horseradish peroxidase (Thermo Fisher Scientific) at a 1:3000 dilution to determine complement activation by measurement of C4b deposition. Data were obtained in at least triplicate and normalized to a well containing serum only (100% complement activation) and background subtracted using a negative control with buffer containing no serum. Plates were washed three times between each step with tris-buffered saline (50 nM Tris pH 8.0, 150 nM NaCl, and 0.05% Tween 20). An EnSight Multimode Plate Reader (PerkinElmer) was used to read the absorbance values at 450 nm for the reaction. Half-maximal inhibitory concentration values (IC_50_) were obtained using the nonlinear variable slope regression fit from GraphPad Prism version 9.5 (https://www.graphpad.com/).

### CP-dependent hemolysis assay

A CP hemolysis assay was performed as previously described (20, 30) to determine if *B. hermsii* FbpC-C WT, H252A, or R248A protected erythrocytes from complement-mediated hemolysis. A serial dilution series of FbpC-C proteins ranging from 1.95 nM to 4 μM were incubated with buffer-exchanged presensitized sheep erythrocytes (Complement Technology) and 2% NHS (Innovative Research). After a 1h incubation at 37 °C, the reaction was clarified *via* centrifugation at 500 × *g* and the absorbance read using an EnSight Multimode Plate Reader (PerkinElmer) at 412 nm. Data were obtained in triplicate and normalized using a positive control with no inhibitor present that represented the 100% hemolysis at 37 °C in 1 h. Background hemolysis was subtracted out using a negative control with only buffer and red blood cells.

### Serum complement-sensitivity assay

Assays were performed as outlined previously (20, 31, 32). Briefly, *B. burgdorferi* strain B314 producing *B. hermsii* FbpC (pAP2), as well as the vector-only control B314 pBBE22*luc*, were grown at 32 °C in 1% CO_2_ in complete BSK-II media with kanamycin at 300 μg/ml to early- to mid-log phase. The cells were then diluted in complete BSK-II media to a final cell density of 1 × 10^6^ cells/ml, then incubated for 1.5 h in 15% NHS (Complement Technology) or equivalent heat inactivated serum as a positive control for survival. Cell survival was assessed by dark field microscopy based on cell motility and membrane damage or lysis.

### Soluble protein inhibition rescue assay

The ability of exogenous *B. hermsii* FbpC-C, and either the R248A mutation or H252A mutation, to rescue serum-sensitive *B. burgdorferi* B314 pBBE22*luc* was determined as in (20). Briefly, recombinant proteins were added at a final concentration of 48 nM, 240 nM, and 1.2 μM, approximately five-fold less than, equal to, and five-fold greater than the concentration of C1r in the human serum sample used. Survival was compared to that of B314 pBBE22*luc* alone and of B314 pAP20, which produces *B. hermsii* FbpC, as described for the serum-sensitivity assays.

### MD simulations

All atom MD simulations for BBK32-C, FbpA-C, FbpB-C, and FbpC-C were carried out with coordinates from their solved crystal structures (PDB IDs: 6N1L, 7RPR, 7RPS, and 8EC3, respectively). Residues not visualized in the final crystal structures due to poor electron density were first modeled with MODELLER v9.1 (83; https://salilab.org/modeller/). OPLS-AA force fields (84) were used with explicit TIP3P water model for all simulations. All atom models for the respective proteins were constructed with hydrogens, and all simulations were performed with Gromacs v 2021.3 (85; https://manual.gromacs.org/2021/download.html). A total of 24,698, 24,283, 23,751, and 33,073 water molecules were used to solvate BBK32-C, FbpA-C, FbpB-C, and FbpC-C systems, respectively. An application of periodic boundary conditions of 10 Å from the edge of the cubic box of dimensions 91.3, 90.8, 90.2, and 100.5 Å, respectively, was implemented. Charge neutralization of the simulation systems for BBK32-C, FbpA-C, FbpB-C, and FbpC-C were undertaken by replacing water molecules with 5 Cl-, 3 Cl-, 1 Cl-, and 2 Cl-ions, respectively. The final systems to be simulated were thus comprised of 76,440 atoms for BBK32-C, 75,355 atoms for FbpA-C, 73,926 atoms for FbpB-C, and 101,858 atoms for FbpC-C. Bond lengths for each of these systems were constrained by LINCS algorithm (86), and all long-range electrostatic interactions were determined using the smooth particle mesh Ewald method (87). Energy minimization was performed with a steepest descent algorithm until convergence (∼1000 steps) with a maximum number of steps set to 50,000. All simulations were performed at 300K. Temperature equilibration was conducted by the isochoric-isothermal NVT ensemble (constant number of particles, volume, and temperature) with a Berendsen thermostat for 100 ps (88). The system was then subjected to pressure equilibration in the NPT ensemble (constant number of particles, pressure, and temperature) for 100 ps using the Parrinello–Rahman protocol (89), maintaining a pressure of 1 bar. Coordinates were saved at an interval of 10 ps, and backbone RMSD, RMSF, radius of gyration, and trajectory analyses were performed with GROMACS programs ‘gmx rms,’ ‘gmx rmsf,’ ‘gmx_gyrate,’ and ‘gmx trjconv’ (90, 91). All simulations were performed for a period of 1 μs and attained convergence based on Cα RMSD and visual inspection. Structural snapshots were extracted from the trajectories at an interval of 100 ps leading to 10,000 snapshots for all simulations. All simulations were performed on a local workstation with CUDA acceleration v11.2 powered by an NVIDIA Quadro GPU with 2560 CUDA compute cores resulting in an average output simulation trajectory of ∼50 ns/day. DCCM and normal mode analyses (all atom and Cα) were performed with Bio3D package (92). Two-dimensional free energy surface generation was performed with reaction coordinates of side chain and main chain distances between the atoms of K1 and K2 site (defined as K1: R248 and K2: K327 for BBK32-C, K1: R264 and K2: K343 for FbpA-C, K1: R309 and K2: N402 for FbpB-C). Two sites were measured for FbpC-C; K1: R248 and K2: K345 and K1: H252 and K2: K345. Coordinates were separated into 100 bins followed by discrete probability distribution calculations according to the equation below, where P_*i*_ is the probability in a particular bin and P_*max*_ the maximal probability, R is the universal gas constant, while temperature (T) is measured in K (298 K) at which all the simulations were performed (83).

$$\Delta G=-RT\;\ln\left(P_{i}/P_{\max}\right)$$

### DCCM analysis

Formal definition of DCCMs is given by the equation below where r_i_ and, r_j_ are the spatial backbone atom positions of the respective ith and jth amino acids (84):

$$C_{\text{ij}}={<\Delta r_{i}\Delta r_{j}>/\left(<\Delta r_{i}^{2}<\Delta r_{j}^{2}>\right)}^{1/2}$$

A time scale is associated with each C_ij_ element, and this time scale corresponds to a subset of contiguous snapshot structures taken from the temporal series of snap-shot structures extracted from the MD trajectory. This timescale (subset of structures) determines the time interval over which the C_ij_ elements are calculated. Positive C_ij_ values result from backbone atom motions between residues i and j that are in the same direction along a given spatial coordinate, while negative C_ij_ values result from backbone atom motions between residues i and j that are opposite in direction along a given spatial coordinate. To normalize such positional fluctuation, all the MD snapshot frames extracted for calculations were superposed to a common frame of reference (*i.e.*, the crystal structure for each protein).

## Data availability

The datasets presented in this study can be found in online repositories. The names of the repository/repositories and accession number(s) can be found below: http://www.wwpdb.org/, 8EC3. All other data are contained within the manuscript.

## Supporting information

This article contains supporting information (29, 31, 32, 35, 37, 81).

## Conflict of interest

The authors declare that they have no conflicts of interest with the contents of this article.

## References

[1] Sharma S., Bhatnagar R., Gaur D. (2020). Complement evasion strategies of human pathogenic bacteria. Indian J. Microbiol..

[2] Lambris J.D., Ricklin D., Geisbrecht B.V. (2008). Complement evasion by human pathogens. Nat. Rev. Microbiol..

[3] Skare J.T., Garcia B.L. (2020). Complement evasion by Lyme disease Spirochetes. Trends Microbiol..

[4] Rosbjerg A., Genster N., Pilely K., Garred P. (2017). Evasion mechanisms used by pathogens to escape the lectin complement pathway. Front. Microbiol..

[5] Anderson C., Brissette C.A. (2021). The brilliance of *Borrelia*: mechanisms of host immune evasion by Lyme disease-causing spirochetes. Pathogens.

[6] Moore S.R., Menon S.S., Cortes C., Ferreira V.P. (2021). Hijacking factor H for complement immune evasion. Front. Immunol..

[7] Marcinkiewicz A.L., Kraiczy P., Lin Y.P. (2017). There is a method to the madness: strategies to study host complement evasion by Lyme disease and relapsing fever spirochetes. Front. Microbiol..

[8] Cleveland D.W., Anderson C.C., Brissette C.A. (2023). *Borrelia miyamotoi*: a comprehensive review. Pathogens.

[9] Coburn J., Garcia B., Hu L.T., Jewett M.W., Kraiczy P., Norris S.J., Lyme (2021). *Disease and Relapsing Fever Spirochetes: Genomics, Molecular Biology, Host Interactions and Disease Pathogenesis*.

[10] Lopez J., Hovius J.W., Bergström S. (2021). *Lyme Disease and Relapsing Fever Spirochetes: Genomics, Molecular Biology, Host Interactions and Disease Pathogenesis*.

[11] Kraiczy P. (2016). Travelling between two worlds: complement as a gatekeeper for an expanded host range of Lyme disease spirochetes. Vet. Sci..

[12] Kraiczy P., Stevenson B. (2013). Complement regulator-acquiring surface proteins of *Borrelia burgdorferi*: structure, function and regulation of gene expression. Ticks Tick Borne Dis..

[13] Rossmann E., Kraiczy P., Herzberger P., Skerka C., Kirschfink M., Simon M.M. (2008). BhCRASP-1 of the relapsing fever spirochete *Borrelia hermsii* is a factor H- and plasminogen-binding protein. Int. J. Med. Microbiol..

[14] Rossmann E., Kraiczy P., Herzberger P., Skerka C., Kirschfink M., Simon M.M. (2007). Dual binding specificity of a *Borrelia hermsii* -associated complement regulator-acquiring surface protein for factor H and plasminogen discloses a putative virulence factor of relapsing fever spirochetes. J. Immunol..

[15] Schott M., Grosskinsky S., Brenner C., Kraiczy P., Wallich R. (2010). Molecular characterization of the interaction of *Borrelia parkeri* and *Borrelia turicatae* with human complement regulators. Infect. Immun..

[16] Hovis K.M., McDowell J.V., Griffin L.T., Marconi R.T. (2004). Identification and characterization of a linear-plasmid-encoded factor H-binding protein (FhbA) of the relapsing fever spirochete *Borrelia hermsii*. J. Bacteriol..

[17] Röttgerding F., Kraiczy P. (2020). Immune evasion strategies of relapsing fever spirochetes. Front. Immunol..

[18] Grosskinsky S., Schott M., Brenner C., Cutler S.J., Simon M.M., Wallich R. (2010). Human complement regulators C4b-binding protein and C1 esterase inhibitor interact with a novel outer surface protein of *Borrelia recurrentis*. PLoS Negl. Trop. Dis..

[19] Grosskinsky S., Schott M., Brenner C., Cutler S.J., Kraiczy P., Zipfel P.F. (2009). *Borrelia recurrentis* employs a novel multifunctional surface protein with anti-complement, anti-opsonic and invasive potential to escape innate immunity. PLoS One.

[20] Booth C.E., Powell-Pierce A.D., Skare J.T., Garcia B.L. (2022). *Borrelia miyamotoi* FbpA and FbpB are immunomodulatory outer surface lipoproteins with distinct structures and functions. Front. Immunol..

[21] Schmidt F.L., Sürth V., Berg T.K., Lin Y.-P., Hovius J.W., Kraiczy P. (2021). Interaction between *Borrelia miyamotoi* variable major proteins Vlp15/16 and Vlp18 with plasminogen and complement. Sci. Rep..

[22] Röttgerding F., Wagemakers A., Koetsveld J., Fingerle V., Kirschfink M., Hovius J.W. (2017). Immune evasion of *Borrelia miyamotoi*: CbiA, a novel outer surface protein exhibiting complement binding and inactivating properties. Sci. Rep..

[23] Stone B.L., Brissette C.A. (2017). Host immune evasion by Lyme and relapsing fever Borreliae: findings to lead future studies for *Borrelia miyamotoi*. Front. Immunol..

[24] Probert W.S., Kim J.H., Höök M., Johnson B.J.B. (2001). Mapping the ligand-binding region of *Borrelia burgdorferi* fibronectin-binding protein BBK32. Infect. Immun..

[25] Fikrig E., Feng W., Barthold S.W., Telford S.R., Flavell R.A. (2000). Arthropod- and host-specific *Borrelia burgdorferi bbk32* expression and the inhibition of spirochete transmission. J. Immunol..

[26] Harris G., Ma W., Maurer L.M., Potts J.R., Mosher D.F. (2014). *Borrelia burgdorferi* protein BBK32 binds to soluble fibronectin via the N-terminal 70-kDa region, causing fibronectin to undergo conformational extension. J. Biol. Chem..

[27] Kim J.H., Singvall J., Schwarz-Linek U., Johnson B.J.B., Potts J.R., Höök M. (2004). BBK32, a fibronectin binding MSCRAMM from *Borrelia burgdorferi*, contains a disordered region that undergoes a conformational change on ligand binding. J. Biol. Chem..

[28] Seshu J., Esteve-Gassent M.D., Labandeira-Rey M., Kim J.H., Trzeciakowski J.P., Höök M. (2006). Inactivation of the fibronectin-binding adhesin gene bbk32 significantly attenuates the infectivity potential of *Borrelia burgdorferi*. Mol. Microbiol..

[29] Hyde J.A., Weening E.H., Chang M., Trzeciakowski J.P., Höök M., Cirillo J.D. (2011). Bioluminescent imaging of *Borrelia burgdorferi in vivo* demonstrates that the fibronectin-binding protein BBK32 is required for optimal infectivity. Mol. Microbiol..

[30] Garcia B.L., Zhi H., Wager B., Höök M., Skare J.T. (2016). *Borrelia burgdorferi* BBK32 inhibits the classical pathway by blocking activation of the C1 complement complex. PLoS Pathog..

[31] Xie J., Zhi H., Garrigues R.J., Keightley A., Garcia B.L., Skare J.T. (2019). Structural determination of the complement inhibitory domain of *Borrelia burgdorferi* BBK32 provides insight into classical pathway complement evasion by lyme disease spirochetes. PLoS Pathog..

[32] Garrigues R.J., Powell-Pierce A.D., Hammel M., Skare J.T., Garcia B.L. (2021). A structural basis for inhibition of the complement initiator protease C1r by Lyme disease spirochetes. J. Immunol..

[33] Lewis E.R.G., Marcsisin R.A., Campeau Miller S.A., Hue F., Phillips A., AuCoin D.P. (2014). Fibronectin-binding protein of *Borrelia hermsii* expressed in the blood of mice with relapsing fever. Infect. Immun..

[34] Brenner C., Bomans K., Habicht J., Simon M.M., Wallich R. (2013). Mapping the Ligand-Binding Region of *Borrelia hermsii* Fibronectin-Binding Protein. PLoS One.

[35] Jumper J., Evans R., Pritzel A., Green T., Figurnov M., Ronneberger O. (2021). Highly accurate protein structure prediction with AlphaFold. Nature.

[36] Curry S. (2015). Structural biology: a century-long journey into an unseen world. Interdiscip. Sci. Rev..

[37] Sadziene A., Wilske B., Ferdows M.S., Barbour A.G. (1993). The cryptic *ospC* gene of *Borrelia burgdorferi* B31 is located on a circular plasmid. Infect. Immun..

[38] Hollingsworth S.A., Dror R.O. (2018). Molecular dynamics simulation for all. Neuron.

[39] Zwier M.C., Chong L.T. (2010). Reaching biological timescales with all-atom molecular dynamics simulations. Curr. Opin. Pharmacol..

[40] Xu Y., Havenith M. (2015). Perspective: watching low-frequency vibrations of water in biomolecular recognition by THz spectroscopy. J. Chem. Phys..

[41] Milano T., Gulzar A., Narzi D., Guidoni L., Pascarella S. (2017). Molecular dynamics simulation unveils the conformational flexibility of the interdomain linker in the bacterial transcriptional regulator GabR from *Bacillus subtilis* bound to pyridoxal 5’-phosphate. PLoS One.

[42] Kufareva I., Abagyan R. (2012). Methods of protein structure comparison. Methods Mol. Biol..

[43] Martínez L. (2015). Automatic identification of mobile and rigid substructures in molecular dynamics simulations and fractional structural fluctuation analysis. PLoS One.

[44] Brooks B., Karplus M. (1985). Normal modes for specific motions of macromolecules: application to the hinge-bending mode of lysozyme. Proc. Natl. Acad. Sci. U. S. A..

[45] Brooks B., Karplus M. (1983). Harmonic dynamics of proteins: normal modes and fluctuations in bovine pancreatic trypsin inhibitor. Proc. Natl. Acad. Sci. U. S. A..

[46] Noguti T., Go N. (1982). Collective variable description of small-amplitude conformational fluctuations in a globular protein. Nature.

[47] Go N., Noguti T., Nishikawa T. (1983). Dynamics of a small globular protein in terms of low-frequency vibrational modes. Proc. Natl. Acad. Sci. U. S. A..

[48] Levy R.M., Srinivasan A.R., Olson W.K., McCammon J.A. (1984). Quasi-harmonic method for studying very low frequency modes in proteins. Biopolymers.

[49] Levitt M., Sander C., Stern P.S. (1985). Protein normal-mode dynamics: trypsin inhibitor, crambin, ribonuclease and lysozyme. J. Mol. Biol..

[50] Henry E.R., Eaton W.A., Hochstrasser R.M. (1986). Molecular dynamics simulations of cooling in laser-excited heme proteins. Proc. Natl. Acad. Sci. U. S. A..

[51] Yamato T., Laprévote O. (2019). Normal mode analysis and beyond. Biophys. Physicobiol..

[52] Alexandrov V. (2005). Normal modes for predicting protein motions: a comprehensive database assessment and associated web tool. Protein Sci..

[53] Ichiye T., Karplus M. (1991). Collective motions in proteins: a covariance analysis of atomic fluctuations in molecular dynamics and normal mode simulations. Proteins.

[54] McCammon J.A., Harvey S.C. (1987). Dynamics of Proteins and Nucleic Acids.

[55] Kormos B.L., Baranger A.M., Beveridge D.L. (2007). A study of collective atomic fluctuations and cooperativity in the U1A-RNA complex based on molecular dynamics simulations. J. Struct. Biol..

[56] Gaieb Z., Morikis D. (2017). Detection of side chain rearrangements mediating the motions of transmembrane helices in molecular dynamics simulations of g protein-coupled receptors. Comput. Struct. Biotechnol. J..

[57] Kuzmanic A., Sutto L., Saladino G., Nebreda A.R., Gervasio F.L., Orozco M. (2017). Changes in the free-energy landscape of p38α MAP kinase through its canonical activation and binding events as studied by enhanced molecular dynamics simulations. Elife.

[58] Berteotti A., Cavalli A., Branduardi D., Gervasio F.L., Recanatini M., Parrinello M. (2009). Protein conformational transitions: the closure mechanism of a kinase explored by atomistic simulations. J. Am. Chem. Soc..

[59] de Jong N.W.M., van Kessel K.P.M., van Strijp J.A.G. (2019). Immune evasion by *Staphylococcus aureus*. Microbiol. Spectr..

[60] Hovingh E.S., van den Broek B., Jongerius I. (2016). Hijacking complement regulatory proteins for bacterial immune evasion. Front. Microbiol..

[61] Herr A.B., Thorman A.W. (2017). Hiding in plain sight: immune evasion by the staphylococcal protein SdrE. Biochem. J..

[62] Dowdell A.S., Murphy M.D., Azodi C., Swanson S.K., Florens L., Chen S. (2017). Comprehensive spatial analysis of the *Borrelia burgdorferi* lipoproteome reveals a compartmentalization bias toward the bacterial surface. J. Bacteriol..

[63] Guo H.B., Perminov A., Bekele S., Kedziora G., Farajollahi S., Varaljay V. (2022). AlphaFold2 models indicate that protein sequence determines both structure and dynamics. Sci. Rep..

[64] Schames J.R., Henchman R.H., Siegel J.S., Sotriffer C.A., Ni H., McCammon J.A. (2004). Discovery of a novel binding trench in HIV integrase. J. Med. Chem..

[65] Dror R.O., Mildorf T.J., Hilger D., Manglik A., Borhani D.W., Arlow D.H. (2015). Structural basis for nucleotide exchange in heterotrimeric G proteins. Science (1979).

[66] Lower S.K., Lamlertthon S., Casillas-Ituarte N.N., Lins R.D., Yongsunthon R., Taylor E.S. (2011). Polymorphisms in fibronectin binding protein A of *Staphylococcus aureus* are associated with infection of cardiovascular devices. Proc. Natl. Acad. Sci. U. S. A..

[67] Greene N.P., Hinchliffe P., Crow A., Ababou A., Hughes C., Koronakis V. (2013). Structure of an atypical periplasmic adaptor from a multidrug efflux pump of the spirochete *Borrelia burgdorferi*. FEBS Lett..

[68] Hognon C., Garaude S., Timmins J., Chipot C., Dehez F., Monari A. (2019). Molecular bases of DNA packaging in bacteria revealed by all-atom molecular dynamics simulations: the case of histone-like proteins in *Borrelia burgdorferi*. J. Phys. Chem. Lett..

[69] Hart T.M., Dupuis A.P., Tufts D.M., Blom A.M., Starkey S.R., Rego R.O.M. (2021). Host tropism determination by convergent evolution of immunological evasion in the Lyme disease system. PLoS Pathog..

[70] Combs M., Marcinkiewicz A.L., Dupuis A.P., Davis A.D., Lederman P., Nowak T.A. (2022). Phylogenomic diversity elucidates mechanistic insights into Lyme Borreliae-Host Association. mSystems.

[71] Lin Y.P., Diuk-Wasser M.A., Stevenson B., Kraiczy P. (2020). Complement evasion contributes to Lyme Borreliae–Host Associations. Trends Parasitol..

[72] Geisbrecht B.V., Bouyain S., Pop M. (2006). An optimized system for expression and purification of secreted bacterial proteins. Protein Expr. Purif..

[73] Scott Samuels D., Drecktrah D., Hall L.S. (2018). Genetic transformation and complementation. Methods Mol. Biol..

[74] Sievers F., Wilm A., Dineen D., Gibson T.J., Karplus K., Li W. (2011). Fast, scalable generation of high-quality protein multiple sequence alignments using Clustal Omega. Mol. Syst. Biol..

[75] Wollweber L. (1998). Antibodies: A Laboratory Manual.

[76] Zwart P.H., Afonine P.V., Grosse-Kunstleve R.W., Hung L.W., Ioerger T.R., McCoy A.J. (2008). Automated structure solution with the PHENIX suite. Methods Mol. Biol..

[77] Karplus P.A., Diederichs K. (2012). Linking crystallographic model and data quality. Science (1979).

[78] Adams P.D., Grosse-Kunstleve R.W., Hung L.W., Ioerger T.R., McCoy A.J., Moriarty N.W. (2002). PHENIX: building new software for automated crystallographic structure determination. Acta Crystallogr..

[79] Adams P.D., Afonine P. v, Bunkóczi G., Chen V.B., Davis I.W., Echols N. (2010). PHENIX: a comprehensive Python-based system for macromolecular structure solution. Acta Crystallogr..

[80] Emsley P., Lohkamp B., Scott W.G., Cowtan K. (2010). Features and development of Coot. Acta Crystallogr..

[81] Mirdita M., Schütze K., Moriwaki Y., Heo L., Ovchinnikov S., Steinegger M. (2022). ColabFold: making protein folding accessible to all. Nat. Methods.

[82] Rushing B.R., Rohlik D.L., Roy S., Skaff D.A., Garcia B.L. (2020). Targeting the initiator protease of the classical pathway of complement using fragment-based drug discovery. Molecules.

[83] Šali A., Blundell T.L. (1993). Comparative protein modelling by satisfaction of spatial restraints. J. Mol. Biol..

[84] Sambasivarao S. v, Acevedo O. (2009). Development of OPLS-AA force field parameters for 68 unique ionic liquids. J. Chem. Theory Comput..

[85] Berendsen H.J.C., van der Spoel D., van Drunen R. (1995). GROMACS: a message-passing parallel molecular dynamics implementation. Comput. Phys. Commun..

[86] Hess B., Bekker H., Berendsen H.J.C., Fraaije J.G.E.M. (1997). LINCS: a linear constraint solver for molecular simulations. J. Comput. Chem..

[87] Essmann U., Perera L., Berkowitz M.L., Darden T., Lee H., Pedersen L.G. (1995). A smooth particle mesh Ewald method. J. Chem. Phys..

[88] Berendsen H.J.C., Postma J.P.M., van Gunsteren W.F., Dinola A., Haak J.R. (1984). Molecular dynamics with coupling to an external bath. J. Chem. Phys..

[89] Parrinello M., Rahman A. (1981). Polymorphic transitions in single crystals: a new molecular dynamics method. J. Appl. Phys..

[90] van der Spoel D., Lindahl E., Hess B., Groenhof G., Mark A.E., Berendsen H.J.C. (2005). GROMACS: fast, flexible, and free. J. Comput. Chem..

[91] Abraham M.J., Murtola T., Schulz R., Páll S., Smith J.C., Hess B. (2015). GROMACS: high performance molecular simulations through multi-level parallelism from laptops to supercomputers. SoftwareX.

[92] Grant B.J., Rodrigues A.P.C., ElSawy K.M., McCammon J.A., Caves L.S.D. (2006). Bio3d: an R package for the comparative analysis of protein structures. Bioinformatics.

